# Comparison of metabolic profiles, regulatory pathways, and quorum sensing inhibition activity of brown mustard seeds (*Brassica juncea L*.) post germination and roasting treatments

**DOI:** 10.1038/s41538-025-00606-5

**Published:** 2025-11-11

**Authors:** Dina S. Ghallab, Mona E. M. Mabrouk, Nourhan G. Naga

**Affiliations:** 1 Department of Pharmacognosy, Faculty of Pharmacy, Alexandria University, Alexandria, Egypt; 2 Botany and Microbiology Department, Faculty of Science, Damanhour University, Damanhour, Egypt; 3 Department of Botany and Microbiology, Faculty of Science, Alexandria University, Alexandria, Egypt

**Keywords:** Biochemistry, Biotechnology, Chemical biology, Microbiology, Plant sciences

## Abstract

While mustard seeds offer an enormous wealth of biologically valuable compounds, the obtainable data unveiling the effect of germination and roasting treatments on the metabolic patterns and quorum-sensing (QS) antagonistic effect is limited and demands further investigation. The current study emphasizes proffering a glimpse of the metabolic fluctuations post mustard seeds germination and roasting treatments through UPLC–MS/MS analysis coupled with chemometrics, and its potential entanglements on disrupting the QS system in *Pseudomonas aeruginosa* PAO1. Correspondingly, 74 chromatographic signals embracing amino acids, phenolic acids, glucosinolates, flavonoids, fatty acids, phospholipids, and terpenoids were chemically characterized. Orthogonal Projection to Latent Structures-Discriminant Analysis (OPLS-DA) models demonstrated perceivable chemical discrepancies among the processed samples, where phenolic acids and flavonoids witnessed a relatively marked rise in germinated and roasted samples compared to raw matches. Nevertheless, aliphatic glucosinolates, primarily sinigrin, glucocheirolin, gluconapin, and progoitrin, along with indole glucosinolates as glucobrassicin, witnessed a moderate elevation with a fold increase (2.5–4.5) in germinated seeds. In contrast, they offered a significant down-regulating tendency following roasting treatment, demonstrating the thermal degradation of glucosinolates into isothiocyanates and canolol. Following germination and roasting treatments, omega-3 fatty acid (linolenic acid and its hydroxylated form) presented a significant upward trend. Most amino acids, primarily serine and tyramine, as well as phospholipids, were continuously diminished throughout the germination and roasting processes. These metabolic shifts were mainly regulated by phenylalanine, tyrosine, and tryptophan biosynthesis, phenylpropanoid biosynthesis, flavonoid biosynthesis, glucosinolate biosynthesis, and glycerophospholipid metabolism pathways. Experimentally speaking, the germinated samples presented the most leading inhibitory effects against pyocyanin production, biofilm formation, twitching, and swarming motility by 75%, 91%, 76%, and 92%, respectively. The OPLS-derived coefficient plot unveiled that the germinated samples were significantly augmented with biologically credited compounds such as syringic acid, syringetin-3-O-glucoside, daidzein, progoitrin, hydroxyglucobrassicin, as well as eicosapentaenoic acid, which might synergistically underlie the QS inhibitory effect. Such integrative work offers meritorious insights into incorporating mustard sprouts and their bioactives in varying nutraceutical and biological applications for circumventing the development of antibiotic resistance.

## Introduction

In microbial communities, numerous bacterial species are known to possess intricate intracellular and intercellular systems that enable them to interact with their surroundings, communicate with neighbouring cells, and function as multicellular units in a process known as quorum sensing (QS)^1^. The foundation of QS is bacteria’s capacity to produce autoinducers (AI), which are low molecular weight chemical signalling molecules released to the surroundings in a cell density-dependent manner, leading to virulence, biofilm formation, and food spoilage^2^. Therefore, efficiently distributing the QS might be a decisive approach to control and stop the expression of foodborne pathogenic bacteria. Rather than eliminating bacteria as conventional sanitisers do, preventing cell-to-cell contact may suppress the expression of specific phenotypes that are less likely to develop microbial resistance^2^. Undoubtedly, plant sources packed with plentiful bioactive compounds with immense significance may afford a decent choice for QS inhibition, especially since there is a growing disapproval of the usage of chemically produced substances in the food sector^1,3^. Nowadays, edible seeds are favoured by consumers for their delicious taste, promising nutritional value and represent a prime source of main nutrients, primarily omega-3 fatty acids, vitamins and minerals and accounting for 6% of total protein consumed worldwide^4^. Among the promising edible seeds fortified with bioactive compounds and increasingly consumed worldwide are mustard seeds.

Mustard seeds (*Brassica juncea L*.), *Brassicaceae* family, are widely cultivated and traditionally consumed for edible oil and condiment production as well as sauces, fermented vegetables industries^5,6^. Mustard seeds, including black, brown/oriental and white/yellow species, were recorded in many Asian countries as traditional food and complementary medicine to manage colds, cough, muscle pain, arthritis, and diabetes^7^. From a chemical aspect, mustard seeds offer an enormous wealth of chemically and biologically valuable compounds as glucosinolates, phenolic acids, flavonoids, anthocyanins, sterols, omega-3 fatty acids and other sulphur-containing compounds^7,8^. This phytochemical wealth has been tightly connected with multiple health benefits as antitumor, antimicrobial, anti-viral, antidiabetic, and anti-inflammatory attributes^9,10^. Even with all these massive pharmacological properties, mustard seeds remain a little-studied drug development resource, though they might merit more investigation for pharmacological uses.

Given the rapid pace of urbanisation and globalisation, different prevailing ways have been introduced and adopted on edible seeds to upgrade the nutritional and functional attributes of seeds via surpassing gut digestibility of balanced macro and micro-nutrients and limiting the bioavailability of anti-nutrients^11,12^. Germination is among the auspicious green modification approaches increasingly adopted on seeds to fruitfully augment valuable compounds endowed with the biological and health benefits of seeds, pursuing the concept of nutritional therapy^13,14^. Importantly, germination, also designated as sprouting, is an emerging biological process of material transformation implying different physiological, biochemical, and sensory manipulations concluding with significant improvements in the functional and nutritional profiles of germinated seeds manifested by the enrichment of the vitamins and minerals, as we as bioactive secondary metabolites throughout the course of germination^15^. On the other hand, roasting is considered one of the most widely utilised pretreatments on edible seeds, which significantly alters the seeds’ microstructure, chemistry, and physical characteristics^16^. As well, roasting seeds positively affects their colour, flavour, and texture, enhancing the recovery of seed oil nutritional and sensory qualities^17^. Importantly, previous reports have indicated that conventional oven roasting pretreatments augment the release of bioactive compounds, primarily tocopherols, lignans, and phytosterols in sesame seeds^16^. Nevertheless, when sesame seeds are improperly roasted, the Maillard reaction can produce dangerous pollutants, including furans and acrylamides, which in turn unfold, degrade, and aggregate proteins as well as cause vitamins to break down negatively affecting the health potential of sesame seeds^16^. Thus, optimising roasting conditions, such as method, temperature, and duration, seems imperative to maintain the nutritional value, bioactive makeup, and overall quality traits of the consumed seeds.

Despite mustard seeds germination and roasting are widely utilised as pretreatment processing strategies to refine the quality attributes of seeds and satisfy the consumers’ demand^18^, there are some aspects still to be elucidated including understanding metabolic patterns and their dynamic distribution following mustard seeds germination and roasting with an ultimate scope for boosting the productivity of a diverse array of bioactive compounds and relating health benefits in a resource-efficient manner. Also, identifying the main metabolic pathways linked with these treatments and their entanglements with the biological features has been overlooked until now.

To fill these gaps, the present investigation incorporated Ultra-high Performance Liquid Chromatography and mass spectrometry (UPLC–MS/MS) metabolomics avenue, followed by multivariate data analysis to provide an insightful qualitative and quantitative description of chemical fingerprints—the metabolome—of mustard seeds post exposure to germination and roasting treatments. Coincidentally, understanding the orchestrating metabolic pathways beyond the chemical shifts in the analysed samples was successfully investigated and monitored. Further, to experimentally investigate the impact of germination and roasting on bioactive compounds enrichment with significant potential on QS inhibition, the different seeds were directly subjected to QS inhibitory screening. Complementarily, the efficacy fingerprints that harmoniously combat and interfere with QS pathways were picked out using bioinformatics tools. Worthily, no relevant report has investigated how seed germination and roasting contribute to the chemical profile and function of mustard seeds. Such integrative work successfully summarises the missing links between different treatments and seeds’ chemical changes, metabolic pathways and biological traits, which is promising as an eco-friendly strategy forward for improving the nutraceutical value of mustard seeds worldwide and prolonging the food products’ shelf life.

## Results and discussion

### Chemical profiling of mustard seeds post-exposure to germination and roasting treatments

The present investigation objectively incorporated UPLC–MS/MS metabolomics avenue to provide an insightful qualitative and quantitative description of chemical fingerprints—the metabolome—of mustard seeds post exposure to germination and roasting treatments.

**Fig. 1:**
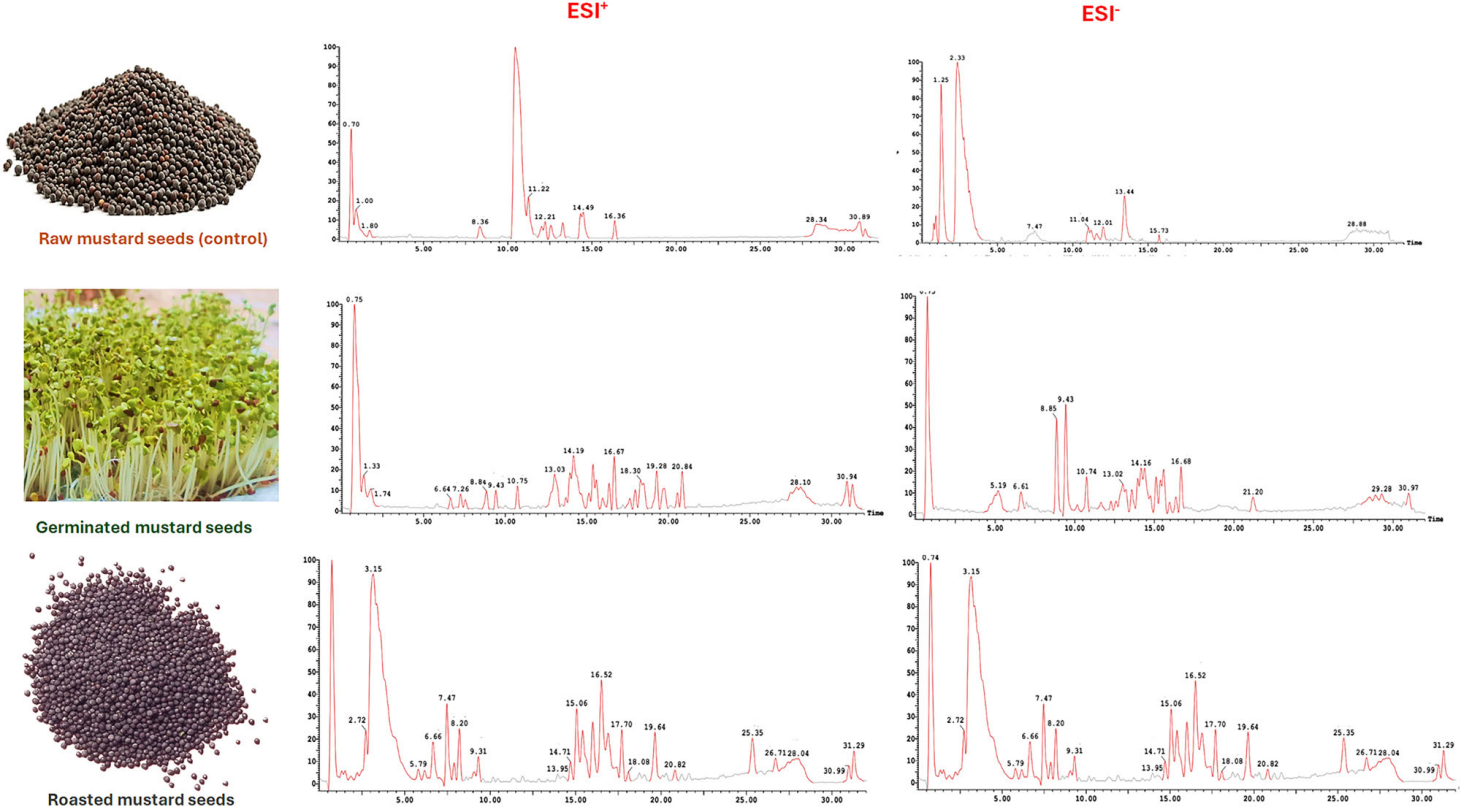
Base peak chromatograms (BPCs) of different mustard seed extracts post-germination and roasting treatments in both positive and negative polarity modes.

Correspondingly, 74 chromatographic signals embracing amino acids, phenolic acids, glucosinolates, thiocyanates, flavonoids, fatty acids, phospholipids, terpenoids and phytosterols were chemically characterised. Table 1 uncovered the full list of the chemically profiled compounds distributed among the differently processed mustard seeds, along with their structural details, primarily retention time, parent ions, unique fragment ions, chemical classes and Human Metabolome Database (HMDB) ID. Also, base peak chromatograms (BPCs) in both positive-ion and negative-ion modes of the different mustard samples, raw, germinated and roasted, are laid out in Fig. 1.

**Table 1:** List of characterised compounds detected in the raw, germinated, and roasted mustard seed extracts through UPLC–MS/MS analysis

No.	Rt (min.)	Identified compounds	Precursor ions	Chemical formulas	Main fragments (Da)	Chemical classes	HMDB ID	References
1	0.76	Allyl isothiocyanate	100.2 M + H	C_4_H_5_NS	72	Isothiocyanates	HMDB0005843	(Song et al.^57^)
2	0.87	Gamma-aminobutyric acid (GABA)	104.2 M + H	C_4_H_9_NO_2_	87-60	Amino acids	HMDB0000112	(Kelley et al.^58^)
3	0.9	Serine	106.2 M + H	C_3_H_7_NO_3_	88-71	Amino acids	HMDB0000187	(P. Zhang et al.^59^)
4	1.12	Sinigrin^a^	398.3 M-H	C_10_H_17_NO_9_S_2_	359-274-195-96	Allyl glucosinolates	HMDB0034070	……
5	1.2	Glycyl valine	175.1 M + H	C_7_H_14_N_2_O_3_	101-76	Dipeptides	HMDB0028854	(Ghallab et al.^42^)
6	1.3	Glutamyl leucine	260.3 M + H	C_11_H_20_N_2_O_5_	132-103	Dipeptides	HMDB0011171	(Ghallab, Shawky, Ibrahim, et al.^60^)
7	1.42	Leucine^a^	132.2 M + H	C_6_H_13_NO_2_	103-86	Amino acids	HMDB0013773	…..
8	1.5	Glucocheirolin	438.4 M-H	C_11_H_21_NO_11_S_3_	332-259-195-135-96-74	Aliphatic glucosinolates	HMDB0000101	(Mohn et al.^61^)
9	1.6	Progoitrin	388.3 M-H	C_11_H_19_NO_10_S_2_	332-259-195-96-74	Aliphatic glucosinolates	HMDB0034071	(Kim et al.^62^)
10	1.7	Gluconapin	372.3 M-H	C_11_H_18_NO_9_S_2_	274-259-195-96-74	Aliphatic glucosinolates	HMDB0000426	(Kim et al.^62^)
11	1.8	Butyl glucosinolate	374.2 M-H	C_11_H_21_NO_9_S_2_	96-95-74	Aliphatic glucosinolates	HMDB0038403	(Hwang et al.^63^)
12	2.1	Indole-3-carbinol	148.2 M + H	C_9_H_9_NO	130-118-91-65	3-alkylindoles	HMDB0005785	(Yu et al.^64^)
13	2.3	Benzyl isothiocyanate	148.2 M-H	C_8_H_7_NS	105-89	Isothiocyanates	HMDB0033969	(Song et al.^57^)
14	2.5	Glucotropaeolin	408.3 M-H	C_14_H_19_NO_9_S_2_	259-195-166-96-74	Aromatic glucosinolates	HMDB0038419	(Hwang et al.^63^)
15	2.6	Gluconasturtiin	421.3 M-H	C_15_H_21_NO_9_S_2_	274-259-195-96-74	Aromatic glucosinolates	HMDB0038423	(Z. Li et al.^65^)
16	2.75	Hydroxyglucobrassicin	463.2 M-H	C_16_H_20_N_2_O_10_S_2_	447-267-169-96	Indole glucosinolates	HMDB0301786	(Hwang et al.^63^)
17	2.9	Phenylethyl isothiocyanate	162.3 M-H	C_9_H_9_NO	120-104	Isothiocyanates	HMDB0256402	(Song et al.^57^)
18	3.2	Glucobrassicin	447.3 M-H	C_16_H_20_N_2_O_9_S_2_	267-169-96	Indole glucosinolates	HMDB0301786	(Hwang et al.^63^)
19	3.4	Hydroxyquinoline	144.3 M-H	C_9_H_7_NO	116-115-85	Hydroquinolones	HMDB0246466	(Ghallab, Nasr, et al.^66^)
20	3.6	Acetyl tyrosine	224.3 M + H	C_11_H_13_NO_4_	182-136-119	Amino acids	HMDB0000866	(P. Zhang et al.^59^)
21	3.9	Salicylic acid-O-hexoside	299.2 M-H	C_13_H_16_O_8_	137-93	Phenolic acids	HMDB0041271	(Mena et al.^67^)
22	4.2	Hydroxybenzoic acid	137.2 M-H	C_7_H_6_O_3_	93	Phenolic acids	HMDB0000500	(Ghallab et al.^68^)
23	4.5	Syringic acid	197.3 M-H	C_9_H_10_O_5_	182-153-167	Phenolic acids	HMDB0002085	(Alberti^69^)
24	4.8	Sinapic acid	225.4 M + H	C_11_H_12_O_5_	179	Phenolic acids	HMDB0032616	(Alberti^69^)
25	5.1	Phenylalanine	166.2 M + H	C_9_H_11_NO_2_	121-104	Amino acids	HMDB0000159	(P. Zhang et al.^59^)
26	5.3	Canolol	181.3 M + H	C_10_H_12_O_3_	165-137-123-94	Phenolic compounds	ChemSpider ID: 33074	(Hu et al.^70^)
27	5.4	Vanillic acid	167.3 M-H	C_8_H_8_O_4_	123-109	Phenolic acids	HMDB0000484	(Mena et al.^67^)
28	5.8	Coumaric acid	163.2 M-H	C_9_H_8_O_3_	135	Phenolic acids	HMDB0001713	(Mena et al.^67^)
29	6.1	Valyl-Phenylalanine	265.2 M + H	C_14_H_20_N_2_O_3_	166-121-118	Dipeptides	HMDB0029134	(Dawczynski et al.^71^)
30	6.2	Sinapine	310.3 M + H	C_16_H_24_NO_5_^+^	251-207-175-147	Phenolic acid derivatives	HMDB0029379	(Guan et al.^72^)
31	6.3	Coumaroyltartaric acid	295.3 M-H	C_13_H_12_O_8_	161-135	Phenolic acids	HMDB0029783	(Guan et al.^72^)
32	6.4	Ferulic acid^a^	193.2 M-H	C_10_H_10_O_4_	179-135	Phenolic acids	HMDB0029200	…..
33	6.6	Dihydrosinapic acid	227.2 M + H	C_11_H_14_O_5_	179-135-161	Phenolic acids	HMDB32616	(Buiarelli et al.^73^)
34	7.2	Tryptamine	161.2 M + H	C_10_H_12_N_2_	145-117	Amino acid derivatives	HMDB0000303	(Schüller et al.^74^)
35	7.4	Ellagic acid	301.2 M-H	C_14_H_6_O_8_	257-229-185	Phenolic acids	HMDB0002899	(Ghallab, Shawky, Ghareeb, et al.^60^)
36	7.6	Chlorogenic acid (3-O-Caffeoylquinic acid)	353.2 M-H	C_16_H_18_O_9_	179-191-135	Phenolic acids	HMDB0003164	(Ghallab & Ghareeb^4,11^)
37	7.8	Astringin	407.4 M + H	C_20_H_22_O_9_	245-134	Stilbenoids	HMDB0002434	(Zhuang et al.^75^)
38	8.2	Catechin	289.3 M-H	C_15_H_14_O_6_	245-205-139	Flavonoids	HMDB0001871	(Dutschke et al.^76^)
39	8.5	Syringetin-3-O-glucoside	507.3 M-H	C_23_H_24_O_13_	346-329-314-261-165-149	Flavonoids	HMDB0304565	(Flamini et al.^77^)
40	8.6	Peonidin-3-O-glucoside	464.3 M + H	C_22_H_23_O_11_	301-286-258-213-201	Flavonoids	HMDB0013689	(Vega et al.^78^)
41	8.8	Methyl Coumarin	161.2 M + H	C_10_H_8_O_2_	147-118	Coumarins	HMDB0032394	(Dugrand et al.^79^)
42	9.2	Luteolin-O-glucoside	449.3 M + H	C_21_H_20_O_11_	285-219-147-73	Flavonoids	HMDB0038468	(Hussain et al.^80^)
43	9.5	Sinalbin A	279.2 M-H	C_12_H_12_N_2_O_2_S_2_	133-124-108	Alkylindoles	HMDB0036650	(Popova & Morra^81^)
44	9.6	Quercetin	301.2 M-H	C_15_H_10_O_7_	255-179-151	Flavonoids	HMDB0005794	(Nada et al.^82^)
45	9.9	Kaempferol^a^	285.3M-H	C_15_H_10_O_6_	331-301-286-194	Flavonoids	HMDB0005801	....…..
46	10.3	Daidzein	253.3 M-H	C_15_H_10_O_4_	237-151-135	Flavonoids	HMDB0003312	(Zhao et al.^83^)
47	10.6	Isorhamnetin	315.3 M-H	C_16_H_12_O_7_	300-285-163	Flavonoids	HMDB0002655	(Alberti^69^)
48	11.1	Feruloyl tryptamine	337.3 M + H	C_20_H_20_N_2_O_3_	195-181-161-137	Phenolic acid derivatives	HMDB0041519	(K. Li & Wang^84^)
49	11.4	Nonanedioic acid (Azelaic acid)	187.3 M-H	C_9_H_16_O_4_	143	Fatty acids	HMDB0000784	(Ghallab et al.^68^)
50	11.7	Hydroxy lauric acid	217.2 M + H	C_12_H_24_O_3_	201-157	Fatty acid “oxylipins”	HMDB0010728	(Griffiths^85^)
51	12.2	Dodecanoic acid (Lauric acid)	201.2 M + H	C_12_H_24_O_2_	157	Fatty acids	HMDB0000638	(Dawczynski et al.^71^)
52	13.4	Carnosic acid	333.3 M + H	C_20_H_28_O_4_	279-246	Terpenoids	HMDB0002358	(Farhadi et al.^86^)
53	13.7	Myristic acid	229.2 M + H	C_14_H_28_O_2_	185-131-75	Fatty acids	HMDB0000806	(Divito et al.^87^)
54	14.8	PE (12:0/16:0)	636.2 M + H	C_33_H_66_NO_8_P	454-436-399-381-257-213	Phospholipids	HMDB0001852	(D. Zhang et al.^33^)
55	15.3	1-palmitoyl-sulfoquinovopyranosylglycerol SQMG (16:0)	555.6 M-H	C_25_H_48_O_11_S	300-255-81	Sulfoglycolipids	HMDB0007853	(Ibrahim et al.^88^)
56	15.8	PG (14:0/14:0)	665.8 M-H	C_34_H_67_O_10_P	648-438-420-227-167	Phosphoglycerols	HMDB0007866	(Ghallab, Nasr, et al.^66^)
57	16.7	LysoPC (16:0)	496.4 M + H	C_24_H_50_NO_7_P	478-259-257-241-213	Phospholipids “Phosphocholines”	HMDB0010382	(Ghallab, Nasr, et al.^66^)
58	17.6	Hydroxy palmitic acid	271.3 M-H	C_16_H_32_O_3_	255-211	Fatty acids” oxylipins”	HMDB0031057	(Gowda et al.^89^)
59	18.2	Stearidionoyl-Glycerol	351.2 M + H	C_21_H_34_O_4_	277-233-162	Fatty acid glycerides	HMDB0244009	(Ray et al.^90^)
60	18.8	Stearidonic acid	277.3 M + H	C_18_H_28_O_2_	255-233-162	Fatty acids	HMDB0006547	(Gowda et al.^89^)
61	19.3	Hydroxy linolenic Acid	293.2 M-H	C_18_H_30_O_3_	277-233-59	Fatty acid “oxylipins”	HMDB0031103	(Ghallab, Ghareeb, et al.^4,11^)
62	19.5	LysoPC 18:3	518.2 M + H	C_26_H_48_NO_7_P	503-277-257-235-239-59	Phospholipids “Phosphocholines”	HMDB0010388	(Ghallab, Ghareeb, et al.^4,11^)
63	19.8	Linolenoyl-glycerol	353.4 M + H	C_21_H_36_O_4_	279-235-59	Lipids (fatty acid glycerides)	HMDB0245187	(Ghallab & Ghareeb^4,11^)
64	20.4	Beta-tocopherol	415.2 M-H	C_28_H_48_O_2_	394-151	Diterpenoids “tocopherols”	HMDB0006335	(Ansolin et al.^91^)
65	20.9	Linolenic acid^a^	277.2 M-H	C_18_H_30_O_2_	235-59	Lipids (fatty acids)	HMDB0003073	……
66	21.3	Linoleoyl-glycerol	355.2 M + H	C_21_H_38_O_4_	281-241-59	Lipids (fatty acid glycerides)	HMDB0245187	(Ghallab & Ghareeb^4,11^)
67	22.3	Eicosapentaenoic acid	303.2 M + H	C_20_H_30_O_2_	259-110	fatty acids	HMDB0001999	(X. Li et al.^92^)
68	23.5	Ricinoleic acid	299.3 M + H	C_18_H_34_O_3_	283-239-126-59	Fatty acids	HMDB0034297	(Choi et al.^93^)
69	23.8	Desmosterol	385.2 M + H	C_27_H_44_O	367-149-163	Sterols	HMDB0002719	(Mo et al.^94^)
70	24.6	Hydroxy arachidonic acid	319.2 M-H	C_20_H_32_O_3_	303-287-259	Fatty acid “oxylipins”	HMDB0060101	(Edpuganti & Mehvar^95^)
71	25.2	Stearic acid	283.4 M-H	C_18_H_36_O_2_		Lipids (fatty acid glycerides)	HMDB0000827	(Ghallab, Shawky, Ibrahim, et al.^96^)
72	26.3	Stigmasterol^a^	413.2 M + H	C_29_H_48_O	399-277-165	Sterols	HMDB0000937	……
73	27.2	Eicosenoic acid	309.2 M-H	C_20_H_38_O_2_	291-265-141-71	Fatty acids	HMDB0002231	(Yang et al.^97^)
74	28.5	Erucic acid	339.2 M + H	C_22_H_42_O_2_	321-295-181	Fatty acids	HMDB0002068	(Crescenzi et al.^98^)

^a^Identified by comparison with authentic references.

Concerning metabolites assignment and following Metabolomics Standards Initiative (MSI) guidelines, 6 signals with numbers 4, 7, 32, 45, 65 and 72 were unambiguously characterised by spectral matching with authentic standards, namely sinigrin, leucine, ferulic acid, kaempferol, linolenic acid and stigmasterol. Further, the remaining compounds were tentatively annotated by spectral comparison of the acquired MS data with our self-established database, referenced spectral databases as Metlin (https://metlin.scripps.edu/auth-login.html) and mzcloud (https://www.mzcloud.org/), as with as relevant literature records.

Notably, using quality control (QC) samples and standard mixing solutions, precision and reproducibility were evaluated to ensure the quality of the generated data.

**Fig. 2:**
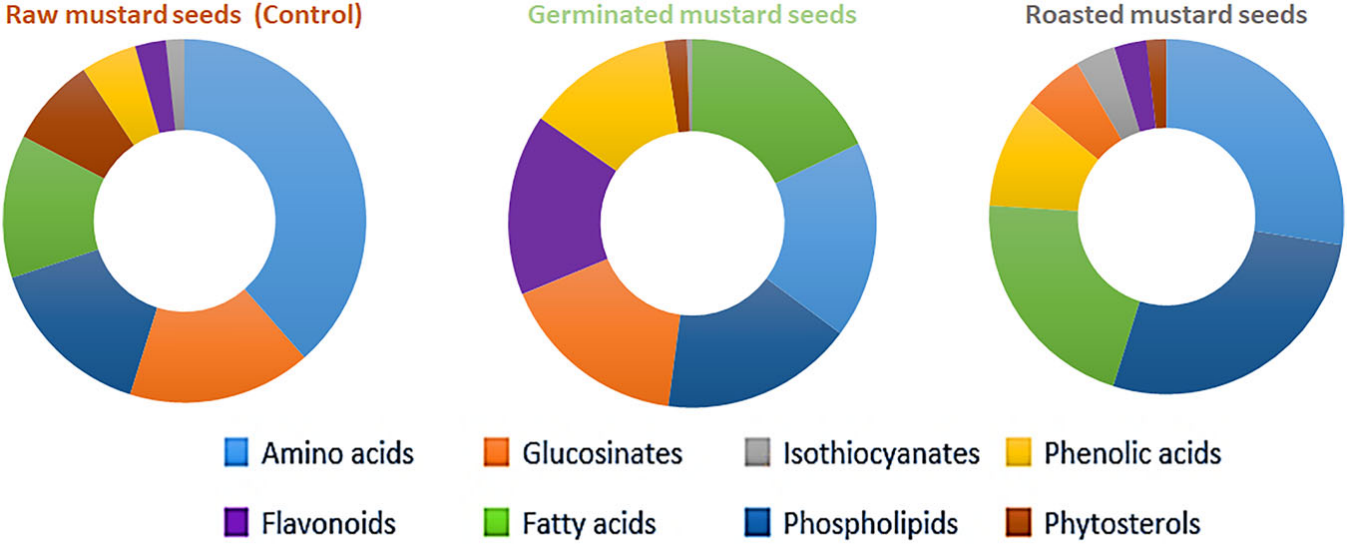
Ring diagrams simply reveal the dynamic distribution of the main chemical classes among the different mustard samples.

In a subsequent set of experiments, the calibration curves of the reference standards stated above were utilised to relatively quantify the chemically profiled classes among various mustard samples, where the relative average content of the main chemical classes in the samples under investigation was calculated and expressed as mg Equivalents/g dry extract, as clarified in Fig. S1. Concisely, Fig. 2 presents the ring diagrams simply revealing the dynamic distribution of the main chemical classes among the mustard samples analysed.

### Chemical discrimination of the differently processed mustard seeds using multivariate statistical analysis

With the intention of handling the large amount of multifaceted chemical data gained from UPLC–MS analysis. Principal component analysis (PCA), as an unsupervised exploratory approach, was preliminarily applied to offer basic insights into the distribution, variability, and grouping patterns among the examined samples in the PC plot.

The four-component PCA model (Fig. S2) effectively separated the mustard samples into two major clusters over the first two orthogonal PCs, accounting for 78.6% of the total variance and guaranteeing discernible fluctuation in their chemical makeup. For clarity, samples of germinated mustard seeds showed distinct chemical variations, as evidenced by their unambiguous discrimination and grouping on the negative direction of PC1, away from the other samples. Conversely, the close clustering of raw and roasted samples across the positive direction of PC1 gently mirrors propinquity in their chemical profile. Further, PC2 represents the intra-chemical differences between raw and roasted samples as they were oppositely dispersed along the PC2 axis.

**Fig. 3:**
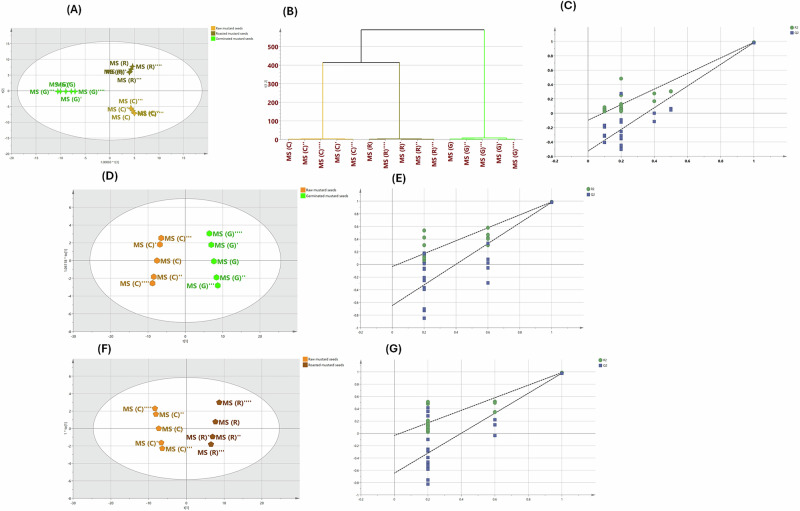
Monitoring the noticeable metabolic changes post germination and roasting treatments using OPLS-DA analysis. **A** OPLS-DA score scatter plot of different mustard samples (raw, germinated and roasted). **B** The respective dendrogram of hierarchical cluster analysis (HCA) by Ward algorithm. **C** The results of the 20 permutation tests. The OPLS-DA models built in pairwise comparisons of different processing treatments as follows (MS (C) & MS (G) (**D**) and (MS (C) & MS (R) (**F**). **E, G** The respective permutation tests.

Considering this, in order to better understand the noticeable metabolic changes post-germination and roasting treatments, a supervised OPLS-DA model was developed. With a good predictive and discriminant capabilities exemplified by the high test parameters; “*R*^2^ (0.984)” and “*Q*^2^ (0.971)”, OPLS-DA score plot (Fig. 3A) demonstrated a discernible separation trend among the examined samples where sprouted samples were grouped together well-distinguishing from the others consulting their striking chemical variations established during germination process while the roasted ones were evidently deviated from the raw group along LV2 positive direction confirming their quite compositional discrepancy. In parallel, two clearly distinguishable clusters were shown by the dendrogram of the examined samples (Fig. 3B). The first cluster corresponded to the sprouted samples, while the remaining samples were clustered together in the second one, assuring their chemical heterogeneity. Additionally, the second cluster showed that the chemical profiles of roasted samples differed noticeably from those of raw ones, as shown by their rather distant sub-clustering.

Of note, the results of the 20 permutation tests (Fig. 3C) demonstrated that the developed model was not overfit.

In order to comprehend the metabolic pathways primarily associated with the differential metabolites and obtain a thorough understanding of metabolite alterations during germination and roasting treatments, the OPLS-DA models were built in pairwise comparisons of different processing treatments in mustard seeds. As highlighted in Fig. 3D, F, a superior separation was illuminated between raw samples (control) and differently processed groups, as follows (MS (C) & MS (G)) and (MS (C). For all pairwise comparisons in the established OPLS-DA models, the *R*^2^Y and *Q*^2^ scores were greater than 0.9, demonstrating the practice models’ viability and accuracy. Permutation tests further revealed that the OPLS-DA models did not overfit (Fig. 3E, G).

### Metabolite patterns and metabolic pathways associated with germination and roasting treatments of mustard seeds

As alluded to above, different modification approaches, mainly germination and roasting, have been increasingly adopted on edible seeds to upgrade their quality attributes and satisfy the consumers’ demand^11^.

**Fig. 4:**
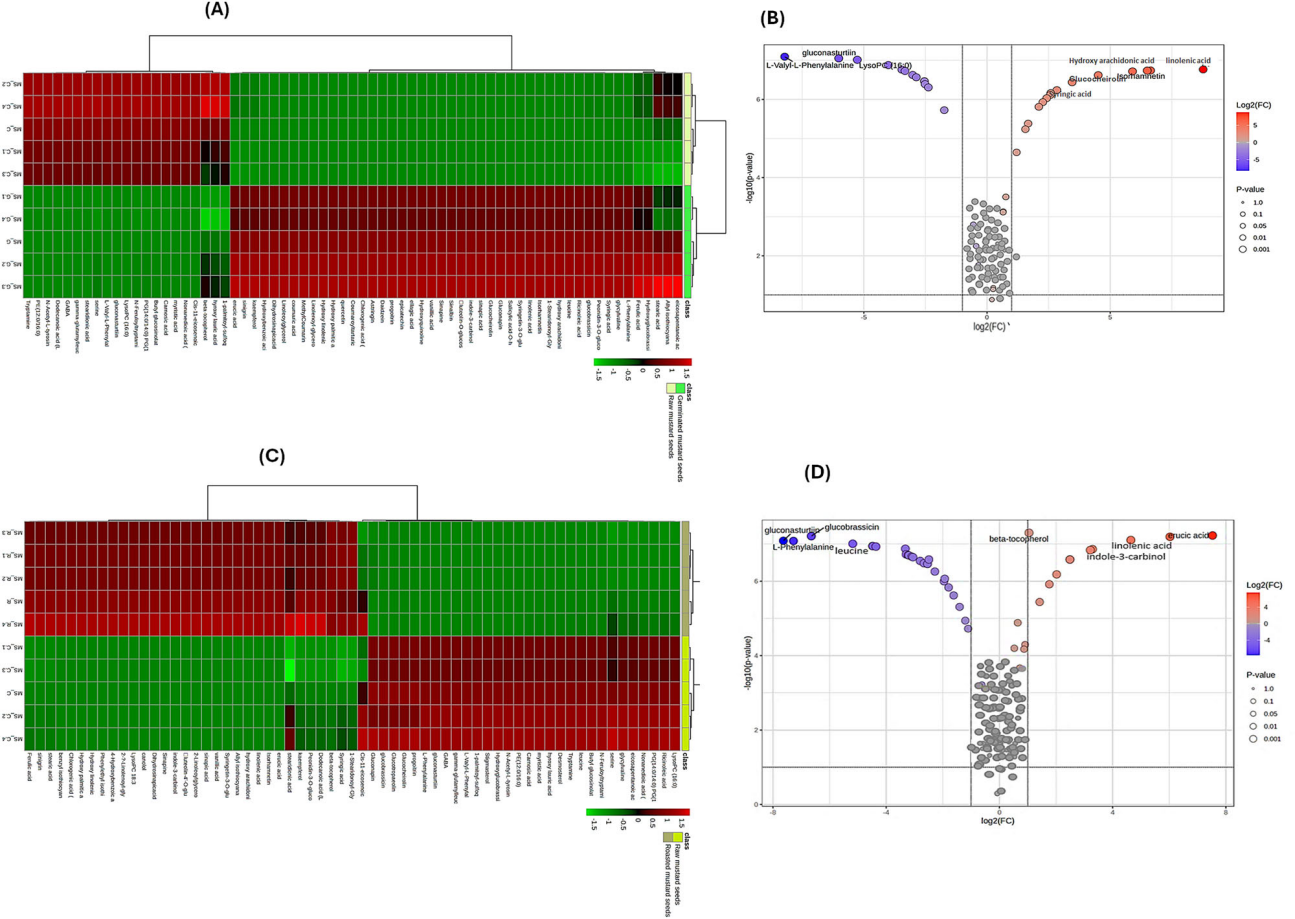
The average relative content of the annotated metabolites among raw, germinated, and roasted samples. Pairwise heatmaps with a color-coded scale grading from red to green representing the relative content of metabolites from high to low where (MS (C) & MS (G) **(A)** and (MS (C) & MS (R) (**C**). **B, D** The corresponding volcano plots constructed to glimpse the differentially cumulated metabolites fulfilling a threshold of *p* < 0.05, VIP > 1 and FC value > 2 or < 0.5 where red dots reflect significant up-regulation while purple ones reflect significant down-regulation of MS (G)/MS (C) or MS (R)/MS (C).

In light of this investigation, pairwise heatmaps with a colour-coded scale grading from red to green representing the relative content of metabolites from high to low (Fig. 4A, C) as well as the corresponding volcano plots (Fig. 4B, D) were constructed to glimpse the differentially cumulated metabolites fulfilling a threshold of *p* < 0.05, Variable Importance in Projection (VIP) > 1 and fold change (FC) value > 2 or <0.5.

For metabolic pathway enrichment analysis, the putative differential metabolites between the comparison groups were then imported into MetaboAnalyst 6.0 (https://www.metaboanalyst.ca/), supported with the Kyoto Encyclopaedia of Genes and Genomes (KEGG) database.

Collectively, supplemental Tables S1 and S2 fully uncover the key metabolic characteristics among the comparison groups, highlighting VIP, FC, and *p*-values as well as the metabolic trends and respective regulatory pathways.

Given this, the differential metabolites were congregated into 7 chemical classes, primarily amino acids, glucosinolates, phenolic acid, flavonoids, fatty acids, phospholipids and phytosterols (Tables S1 and S2). An overview of the primary metabolites’ shifts and associated metabolic pathways during mustard seed germination and roasting is given in the next subsections.

### Amino acids

As alluded to above, mustard seeds’ high protein content and perfectly balanced amino acid makeup majorly contribute to their nutritional benefits^7^.

In the current investigation, among the comparison samples, amino acids constituted one of the largest proportions of differential metabolites (Tables S1 and S2). For the sake of clarity, the relative abundance of most amino acids as GABA, serine, tyramine, acetyl tyrosine and glutamyl leucine was continuously diminished throughout the germination and roasting processes (versus the raw (unprocessed) group) inferring that amino acids are broken down and exploited in multiple physiological functions during treatments, such as energy supply required for seedling growth and development, immune modulation and antioxidant potential^19^. However, phenylalanine and leucine witnessed a noticeable upregulating trend compared with that in raw seeds during the germination process, as they are essential amino acids serving as building blocks of proteins consumed during processing treatments^20^. Also, this rise is the result of activation of proteolytic enzymes, which break down the stored proteins into their smaller building blocks like phenylalanine and leucine, significantly improving the amino acid profile in the germinated seeds, making it a more nutritious food source^20^.

Interestingly, this pattern is positively consistent with other lines of data which experimentally demonstrated that germination augments essential amino acids content in brown rice, wheat and pumpkin seeds, while roasting can lead to a remarkable decline in the amino acids content due to the thermal degradation of some amino acids at high temperatures^4,21^.

**Fig. 5:**
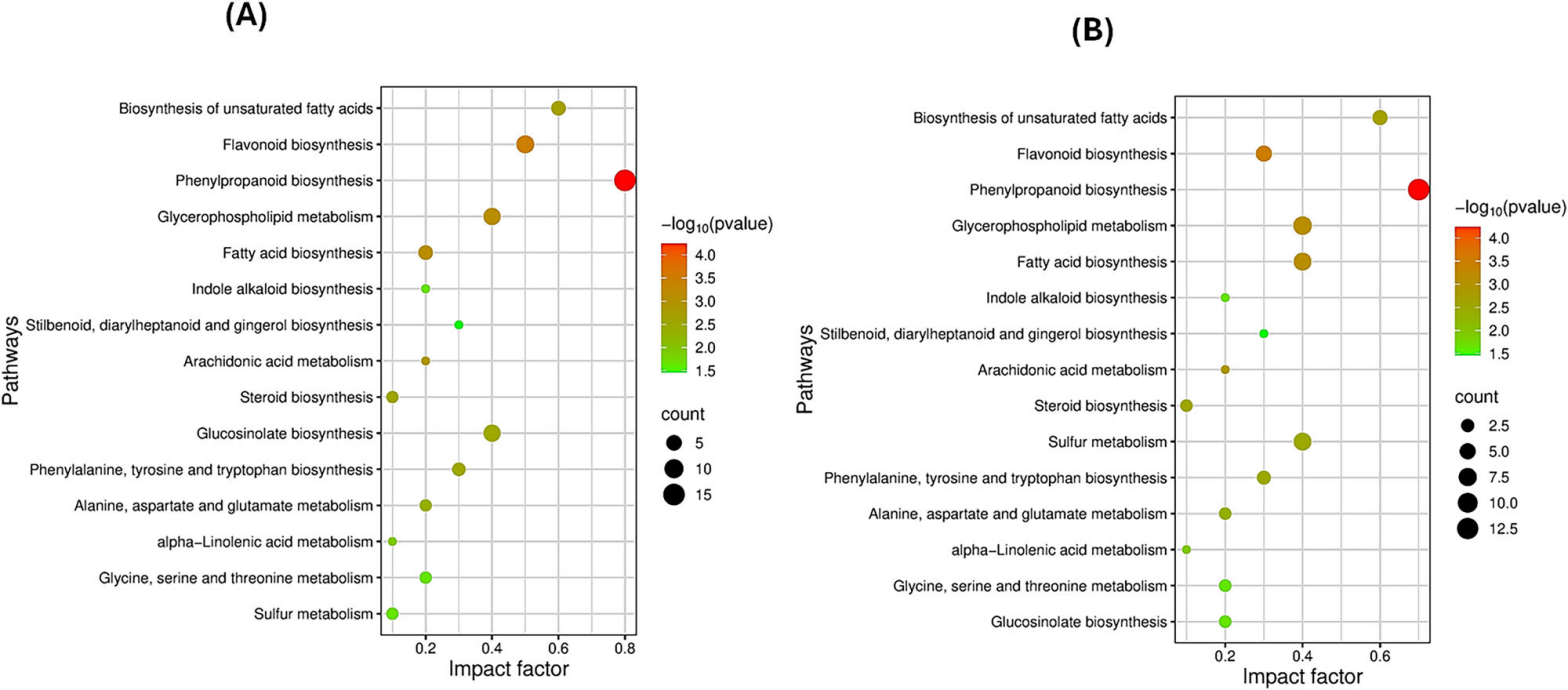
Investigating the metabolic pathway enrichment analysis associated with germination and roasting treatments. KEGG enrichment analysis for the putatively differential metabolites of each pairwise comparison where (MS (C) & MS (G) (**A**) and (MS (C) & MS (R) (**B**).

In comparing germinated and roasted mustard seeds with raw ones, these differentially expressed amino acids were primarily enriched in the three metabolic pathways, namely phenylalanine, tyrosine and tryptophan biosynthesis, alanine, aspartate and glutamate metabolism, as well as glycine, serine and threonine metabolism (Fig. 5A, B).

### Phenolic acids

Mustard seeds offer a powerhouse of valuable antioxidant phenolic compounds with a significant potential in preventing long-term conditions like cancer, heart disease, autoimmune diseases, and neurological diseases^5^.

As listed in Tables S1 and S2, compared with the unprocessed samples, variations in phenolic acid content post-germination and roasting treatments were discernible. For clarity, a striking accumulation of phenolic acids, mainly syringic acid, coumaric acid, hydroxybenzoic acid, vanillic acid, sinapic acid, chlorogenic acid and salicylic acid-O-hexoxide in the sprouted sample with approximately 6-fold relative increments compared with raw matches (Fig. 4A, C), suggesting that the germination process enhances the release of bound phenolics and activates their biosynthetic pathways. This trend favourably aligns with earlier research that showed that germination significantly raised the seed sprouts’ total phenolic content over time, up to 6.8 times higher than the initial concentration of the corresponding raw ones^11,22^. In parallel, following roasting treatment, some phenolic acids and their derivatives, mainly hydroxybenzoic acid, vanillic acid, sinapic acid and sinapine, experienced a noticeable increasing trend compared with raw unprocessed samples as roasting might increase the availability of bound phenolic chemicals from the raw seeds by dissolving their cell walls. Notably, these results concur with earlier studies, which experimentally proved that roasting enhances the total phenolic content and antioxidant activities of whole grains, coffee beans, and cashew nuts^23,24^.

Altogether, as illustrated in Fig. 5, our results indicated that phenylpropanoid biosynthesis is one of the highly changeable metabolic pathways modulated during the germination and roasting process and tightly linked with the notable changes in the overall phenolic content.

### Flavonoids

Relatedly, flavonoids and their derivatives represented one of the putatively differential metabolites across the pairwise comparisons in the current work.

Our study mainly demonstrated perturbations in flavonoid biosynthesis, and these changes are primarily reflected in a noticeable difference in the flavonoid content between the sprouted samples and the raw ones. Herein, a marked rise in the relative abundance of different flavonoids namely isorhamnetin, epicatechin, luteolin-O-glycoside, syringetin-O-glucoside, quercetin, daidzein, peonidin-O-glucoside, and kaempferol with a fold change range from 3 to 6 in the germinated group compared with the raw controls was observed signifying the positive impact of germination in augmenting valuable flavonoid compounds endowed with the biological and health benefits of sprouted seeds pursuing the concept of nutritional therapy (Tables S1 and S2). Worth mentioning, these metabolic perturbations in flavonoids and their respective metabolic pathway, flavonoid biosynthesis, came in line with previous records that experimentally proved that germination fruitfully improved flavonoid and lignan accumulation in pumpkin seed sprouts in a time-dependent manner^4^. Further, another study has pointed out that germination dramatically raised the flavonoid level of red kidney bean sprouts up to 24% compared with raw, ungerminated beans^11^.

Conversely, no significant variation in the flavonoid content was observed in the roasted samples compared with the germinated and raw ones. Of note, few representative examples of flavonoids, namely isorhamnetin, luteolin-O-glycoside, and syringetin-O-glucoside, showed a notable upward trend throughout the roasting process. Consistent with earlier records, it was found that roasting at 150 °C for 15 min was optimal for maintaining the stability of flavonoid or even enhancing the levels of glycosylated flavonoids in faba leaves^25,26^.

### Glucosinolates

Glucosinolates serve as a large structurally and functionally varied sulphur-containing class of natural products predominantly found in mustard seeds^9^.

As data shown (Fig. 5), one of the great changeable metabolic pathways perceived in processed mustard seeds was associated with glucosinolate biosynthesis and sulphur metabolism.

In our study, it was observable that the relative contents of glucosinolates moderately increased over the course of mustard seed germination; however, they afforded a significant down-regulation tendency as roasting progressed. In a more specific approach, aliphatic glucosinolates, primarily sinigrin, glucocheirolin, gluconapin, and progoitrin, as well as indole glucosinolates, such as glucobrassicin, witnessed a moderate elevation with a fold increase (2.5–4.5) in germinated seeds compared with the raw ones. While the aromatic glucosinolates were detected in lesser amounts in sprouted samples compared with the raw ones. This slight elevation or even decline of glucosinolates during germination might be due to the enzymatic breakdown (myrosinase) and utilisation of glucosinolates mainly stored in the seeds as a source of energy and nitrogen for the developing seedling during germination^27^. Further, the dilution effect means that as the seed germinates and the seedling grows, the initial high concentration of glucosinolates in the seed is diluted by the increasing volume of the developing plant tissues. Parallel with a leading study, which dissected that glucosinolate content of *Arabidopsis thaliana* seeds experienced an initial dramatic decline during the seedling development, followed by a slight elevation upon emergence of the rosette leaves^28^. These fluctuations in glucosinolate profiles indicated simultaneous functioning of biosynthesis and turnover reactions, with an initial surplus of biosynthesis followed by a period in which turnover outweighs total biosynthesis.

On the other hand, a marked drop of glucosinolates in the roasted samples compared with raw controls was noticed, demonstrating the thermal degradation of glucosinolates during the roasting process into other compounds, including isothiocyanates, nitriles, and others^29^. These breakdown products, exemplified by allyl isothiocyanate, benzyl isothiocyanate, and canolol, were significantly detected in roasted samples compared with others. Of note, different glucosinolates may degrade at varying rates during roasting, with some being more stable than others. Astonishingly, this degradation is accompanied by the release of different volatile compounds, as nitriles and isothiocyanates majorly contribute to the flavour profile of the roasted food. Worthily, our findings are in good harmony with previously published data^29,30^.

### Fatty acids and their derivatives

In the present investigation, fatty acids, especially unsaturated and hydroxylated forms, were found to be significantly elevated in the roasted and germinated samples and directly implicated in the biosynthesis of the unsaturated fatty acids metabolic pathway. Notably, following germination and roasting treatments, omega-3 fatty acid (linolenic acid and its hydroxylated form) presented a significant upward trend compared with unprocessed samples (Tables S1 and S2). In this investigation, most saturated fatty acids, such as azelaic acid, lauric acid, myristic acid, and eicosenoic acid, were majorly detected in the raw seeds. In the same context, two long-chain saturated fatty acids, such as erucic acid and stearic acid, were found to be overexpressed in the roasted group compared with others. This pattern was in harmony with previously published studies, which showed that the overall fatty acid content decreases during seed germination, which is possibly due to the enzymatic breakdown (lipase) of stored fatty acids and glycerides in a process called beta-oxidation to supply energy and building blocks for new cell structures^17^. Also, another leading report demonstrated that moderate roasting of almond (*Prunus dulcis*) kernel oil at 180 °C for 10–20 min increased levels of unsaturated fatty acids (linolenic and elaidic acids) as well as saturated fatty acids (stearic acid)^31^. However, roasting at a higher temperature (200 °C) for a longer period of time (30 min) may cause fatty acids, particularly unsaturated fatty acids, to break down^31^.

Equally important, our findings demonstrated that fatty acid glyceride alterations during germination were more significant compared to roasted seeds. This metabolic shift in lipid profile with an increase in linoleoyl and linolenoyl glycerols (specifically mono- or di-acylglycerols) is normal during seed germination, and linked to the breakdown of triacylglycerols, which are the primary storage lipids in seeds, into simpler lipid molecules like monoacylglycerols by lipolytic enzymes such as lipase. This process, principally regulated by the glycerolipid metabolism pathway, provides energy and building blocks for the developing seedling, as well as being involved in membrane repair during germination.

### Phospholipids

A number of physiological processes that control plant growth and development, as well as cellular reactions to changes in the environment, are known to be regulated by phospholipids, which are the essential structural elements of plant cell membranes^32^.

As dissected in Tables S1 and S2, three phospholipid classes, namely lysophosphatidylcholines (lysoPC), phosphatidylethanolamines (PE) and phosphatidylglycerol (PG), were picked out as the differentially modulated lipid species during the germination and roasting processes. These lipid species shared a similar regulatory trend across both processes, where they offered a significant downward shift in their overall content with respect to the raw seeds, demonstrating the membrane phospholipids remodelling and their multifaceted functions in the formation of cell membrane architecture and regulating signalling transduction during embryonic development^19^. Importantly, water imbibition during germination activates enzymes like phospholipases. These enzymes break down complex phospholipids into simpler molecules, such as free fatty acids and diacylglycerol^28^.

Further, the heat from roasting can cause the phospholipids to degrade into smaller compounds, such as free fatty acids^33^. Also, phospholipids often contain polyunsaturated fatty acids (PUFAs), which are highly susceptible to oxidation. The intense heat of roasting accelerates this oxidative degradation, causing the fatty acid tails to break down and form new volatile compounds that contribute to roasted flavour^34^. Further, Phospholipids that contain an amino group (e.g., phosphatidylethanolamine) can participate in the Maillard reaction, which degrades the phospholipids and influences the flavour profile. Studies on flaxseed and soybeans confirm that phosphatidylethanolamine (PE) is especially vulnerable to degradation via the Maillard reaction during roasting^35^.

This leads to a net decrease in the overall phospholipid content in roasted seeds. Worthily, these findings matched with previously reported investigations^36,37^.

In conclusion, KEGG pathway analysis (Fig. 5). revealed that these metabolic shifts were directly linked to the glycerophospholipid metabolism pathway.

### Terpenoids and phytosterols

In our investigation, compared with raw seeds, it was apparent that the relative content of phytosterols, such as stigmasterol and desmosterol, was significantly depressed with the seed roasting. However, no significant shift was observed in the germination. Relatedly, a marked drop of carnosic acid in both processed groups compared with raw controls was noted.

Essentially, there was a noticeable decline in beta-tocopherol concentration in roasted samples compared with the others, as tocopherols are susceptible to heat and might be thermally degraded over roasting treatment. This trend matches a previous report that illustrated that the total tocopherol content and the antioxidant capacity exhibit a notable decrease after cocoa beans roasting^38^.

These metabolic perturbations stated above might be associated with ubiquinone and other terpenoid-quinone biosynthesis pathways, as clarified in Fig. 5.

To sum up, the systematic mapping of the key differential metabolites to their respective metabolic pathways represented the first effort to thoroughly investigate the chemical pattern and the associated regulatory pathway network during mustard seed germination and roasting.

### In vitro screening of different mustard samples for microbial growth and quorum-sensing inhibitory activities

#### Antibacterial screening

**Fig. 6:**
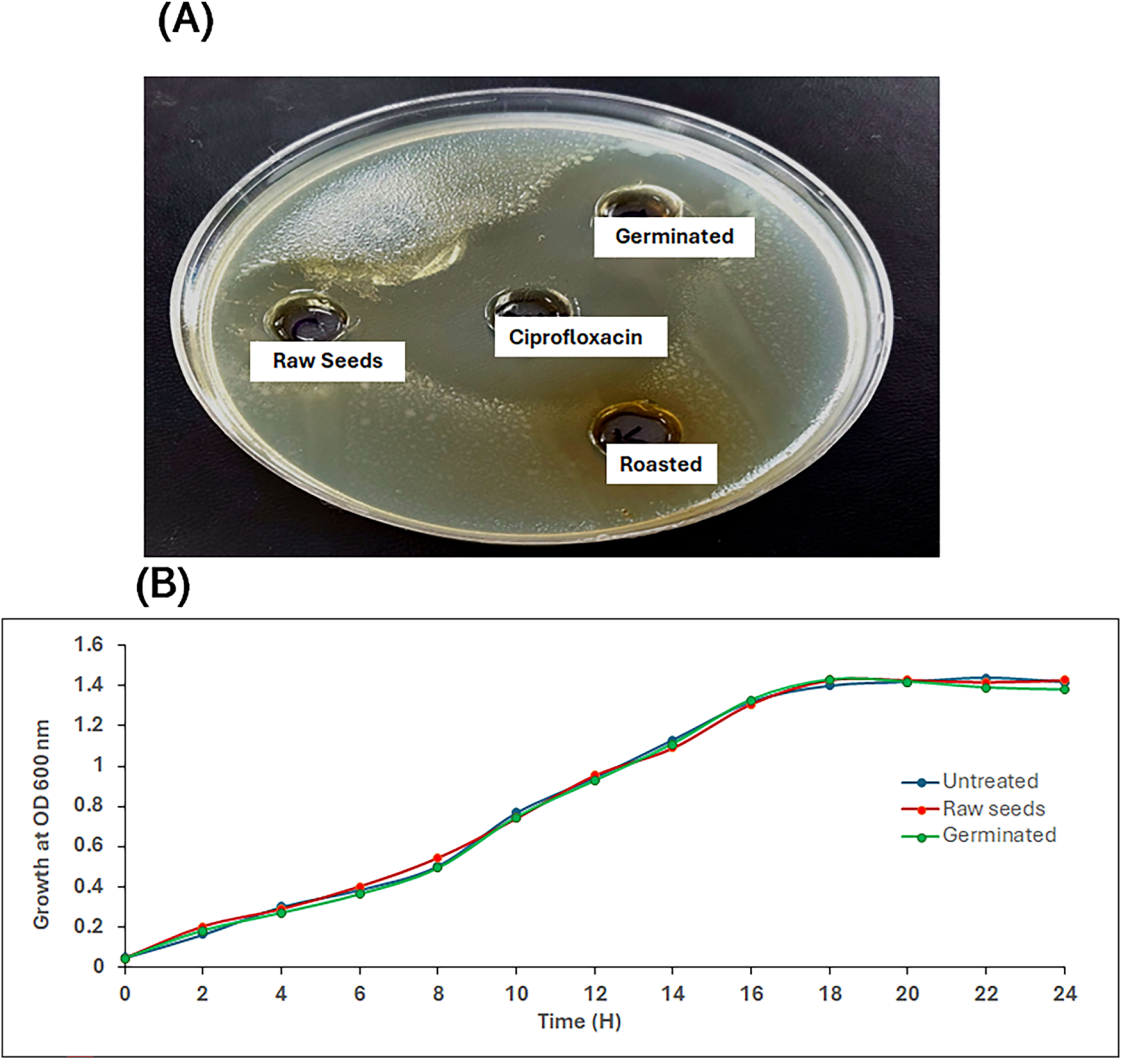
Antibacterial activity assay and determination of sub-MIC effect on bacterial growth. **A** The antibacterial activity of raw, germinated, and roasted mustard seed extracts against *P. aeruginosa* PAO1. **B** The growth curve of PAO1 in the presence and absence of ½ MIC of control and germinated seeds.

All extracts of mustard seeds, raw, germinated and roasted, were screened against *P. aeruginosa* PAO1. The germinated and control seeds showed prominent antibacterial activity, with inhibition zones of 30 and 26 mm, respectively, compared to 34 mm for the positive control, ciprofloxacin. The remaining samples showed no detectable activity (Fig. 6A).

### MIC determination and sub-MIC effects

MIC testing revealed that control and germinated seeds had MICs of 625 and 312 μg/mL, respectively. Growth curve analysis at 312 and 156 μg/mL (½ MIC) for control and germinated seeds, respectively, showed no significant effect on the growth rate of *P. aeruginosa* PAO1 compared to untreated controls (Fig. 6B).

### Evaluation of anti-virulence properties

#### Pyocyanin inhibition

**Fig. 7:**
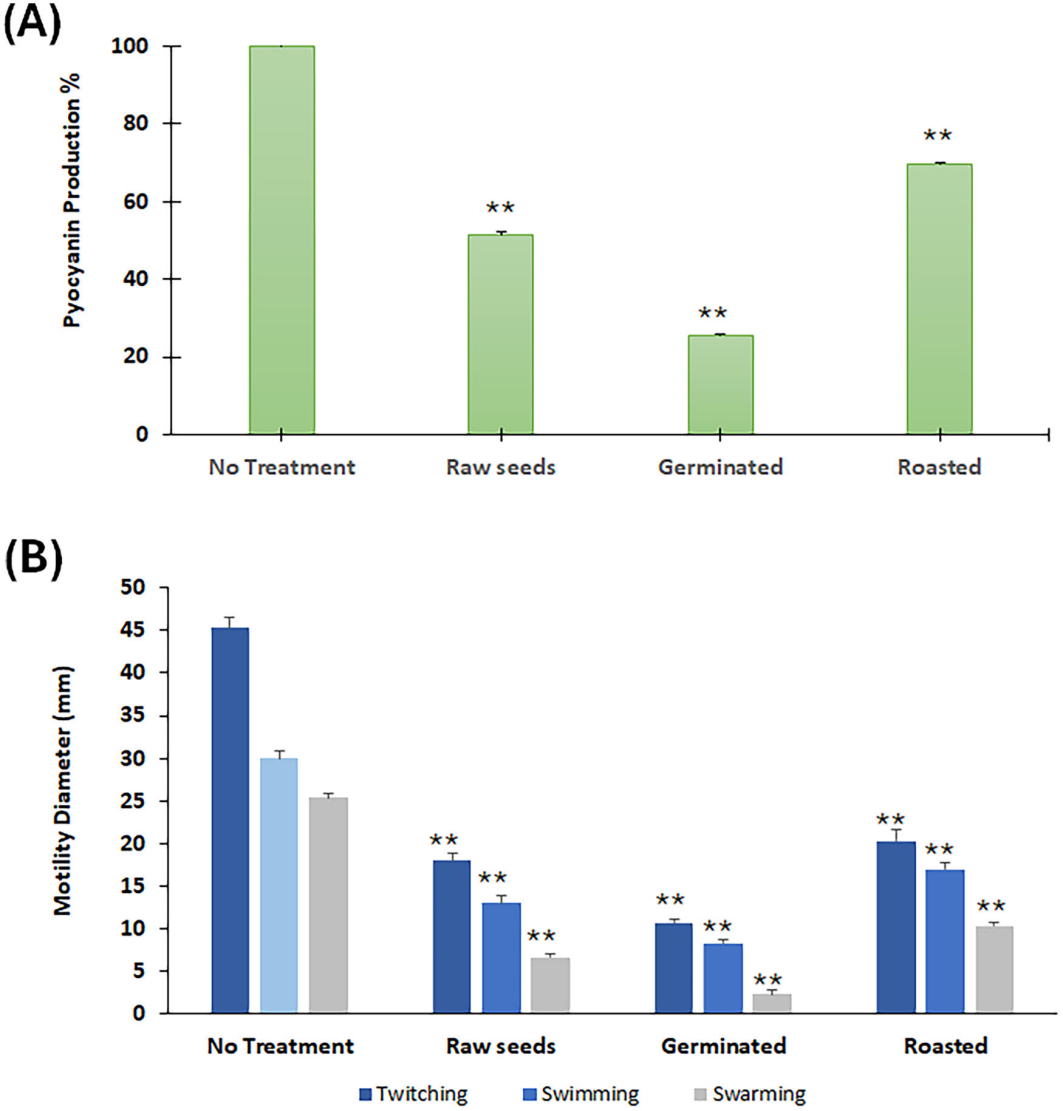
Pyocyanin inhibition assay. Effect of raw, germinated, and roasted mustard seed extracts on pyocyanin production (**A**) and twitching*,* swarming, and swimming motility (**B**) in *P. aeruginosa* PAO1 compared to the untreated cultures. Error bars represent SD (*n* = 3), **p ≤ 0.01.

The control, germinated, and roasted mustard seeds significantly (*p* ≤ 0.01) reduced pyocyanin production. The germinated seeds exhibited the highest inhibition activity by 75% compared to the contro,l and roasted seeds inhibited by 49 and 30%, respectively (Fig. 7A).

### Motility inhibition

The tested mustard extracts caused significant (*p* ≤ 0.01) reductions in all motility forms of *P. aeruginosa* PAO1, where the germinated samples exhibited the highest potency among all. The twitching motility was suppressed by 76%, 60%, and 56% by germinating, control, and roasted mustard seeds, respectively. The swimming motility was inhibited by 73%, 57%, and 43% by germinated, control, and roasted mustard seeds, respectively (Fig. 7B). The swarming was reduced by 92%, 72%, and 60% by germinating, control, and roasted mustard seeds, respectively (Fig. 7B).

### Biofilm formation inhibition

**Fig. 8:**
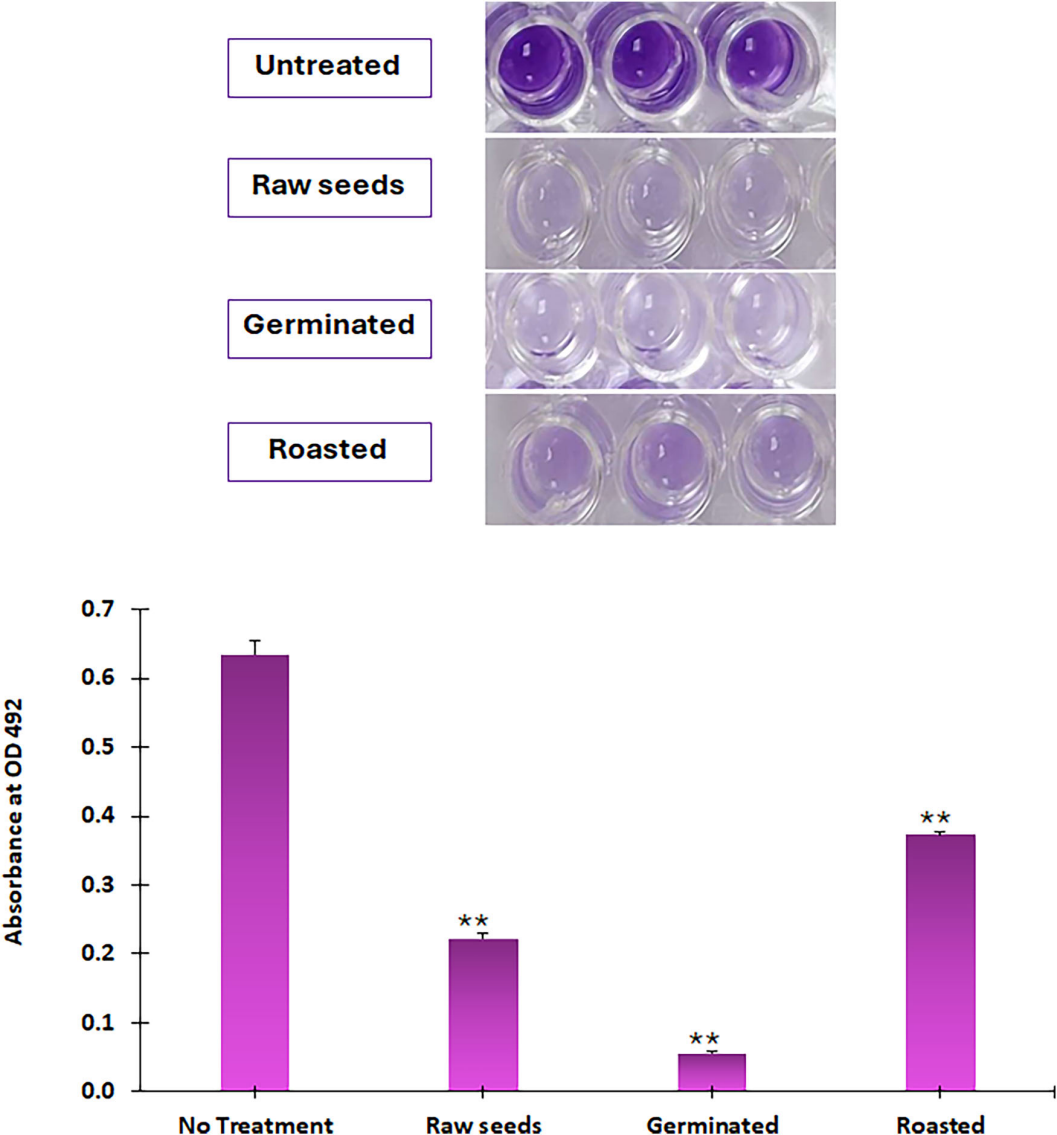
Correlation analysis for pinpointing the efficacy constituents beyond quorum sensing inhibition in the different mustard samples using OPLS analysis. **A** OPLS biplot of the investigated mustard seed extracts in relation to quorum sensing inhibitory activity. **B** Correlation analysis between bioactive.

All seed extracts suppressed biofilm formation significantly (*p* ≤ 0.01). The germinated seeds showed the highest inhibition activity by 91%. The roasted and control seeds showed less inhibition activity by 41% and 65%, respectively (Fig. 8B).

The above findings collectively indicated the profound impact of germination in enhancing the nutritional and biological qualities of seeds through augmenting the potentially bioactive compounds endowed with the control or prevention of microbial infections through quorum-sensing antagonism prospect. In contrast, roasting negatively affects the functional attributes of mustard seeds through degrading some heat-sensitive bioactive compounds, such as glucosinolates, some flavonoids, vitamins, and omega-3 fatty acids. On the positive side, roasting can enhance flavour and aroma profile due to the Maillard reaction and other chemical changes, as well as potentially improve the digestibility of certain compounds by breaking down some antinutrient barriers^39^.

### Correlation analysis for pinpointing the efficacy constituents beyond quorum-sensing inhibition in the different mustard samples using multivariate analysis

With an intention to facilitate the biological interpretability of the previously examined data, a predictive response model, orthogonal projection to latent structures (OPLS), was implemented to track potential bioactive metabolites that work in concert to mediate the inhibitory mechanisms against quorum sensing.

**Fig. 9:**
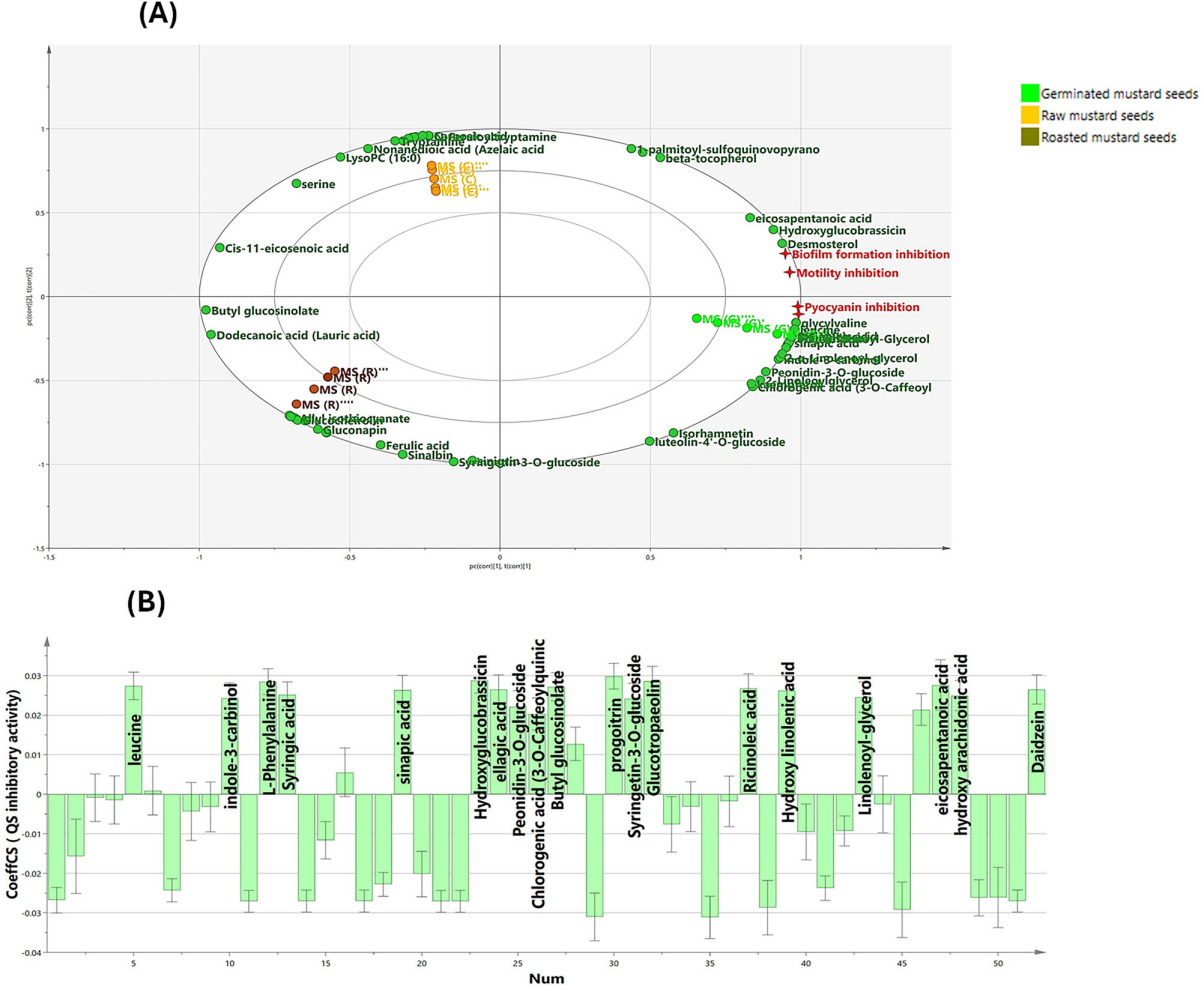
Correlation analysis for pinpointing the efficacy constituents beyond quorum sensing inhibition in the different mustard samples using OPLS analysis. (**A**) OPLS biplot of the investigated mustard seed extracts in relation to quorum sensing inhibitory activity. (**B**) Correlation analysis between bioactive compounds existing in the biologically active samples and QS inhibitory efficacy.

The OPLS biplot (Fig. 9A), evincing 71.3% of the overall variance, manifestly distinguished among the different mustard samples: raw, germinated, and roasted ones that possessed different QS inhibitory potentials, where germinated samples were all congregated across the positive direction of latent variable 1 (LV1), spatially correlated to the QS inhibitory activity (*Y*-variables), reinforcing the prior biological observations (refer to Evaluation of anti-virulence properties section). Whilst roasted samples were projected far away, the *Y*-variables exemplified by pyocyanin, biofilm formation, and motility inhibitory properties that directly reflect ant-QS potential.

Following that, to enhance our biological relevance for the efficacy metabolites that might be beyond the observed QS inhibitory activities, the OPLS-derived coefficient plots were further integrated. Inspection of the OPLS-derived coefficients plot (Fig. 9B) delineated that persuasive bioactive phenolic compounds, mainly syringic acid, sinapic acid, ellagic acid, chlorogenic acid (3-O-Caffeoylquinic acid), peonidin-3-O-glucoside, syringetin-3-O-glucoside, and daidzein, well-praised with their remarkable antimicrobial potential and plentifully existing in the germinated samples, were likely ascribed to the noteworthy QS antagonistic effect. Equally essential, some dominating glucosinolates, namely butyl glucosinolate, progoitrin, hydroxy glucobrassicin, and glucotropaeolin, credited with having a promising antimicrobial action against a vast range of pathogens, might significantly contribute to the observable QS inhibitory potential. Besides this, the relative prevalence of some fatty acids and their derivatives, such as eicosapentaenoic acid, ricinoleic acid, hydroxy linolenic acid, and linoleoyl-glycerol, along with some functional amino acids, particularly leucine and phenylalanine, in raw and sprouted samples might add significant value to the QS antagonism.

Worthily, the gathered findings are consistent with earlier reports uncovering the promising QS inhibitory potential of phenolic acids against foodborne bacterial strains, mainly *Salmonella enterica* serovar Montevideo and *Aeromonas hydrophila*^40^. Relatedly, some compelling evidence has revealed that flavonoids can profoundly disrupt quorum sensing (QS) in certain bacteria via interfering with QS receptors, reducing the production of virulence factors and biofilm formation^40^. Some leading studies have reported the anti-quorum-sensing activity of fatty acids, particularly unsaturated ones, through potentially reducing bacterial virulence and biofilm formation^41^.

These bioactive compounds pinpointed above and considerably augmented in mustard sprouts may offer promise as potential chemical candidates for QS antagonism and combating antibiotic resistance.

In summary, inspired by the long-standing use of mustard seeds credited with their nutritive and biological properties as traditional food and complementary medicine, the present work provides an insightful qualitative and quantitative description of chemical fluctuations of mustard seeds post exposure to germination and roasting treatments and their possible QS antagonistic entanglements. The metabolic analysis has figured out that these metabolic shifts among the mustard samples were mainly regulated by phenylalanine, tyrosine and tryptophan biosynthesis, phenylpropanoid biosynthesis, flavonoid biosynthesis, glucosinolate biosynthesis, and glycerophospholipid metabolism pathways. Regarding QS inhibitory screening, the germinated samples augmented with bioactive compounds exhibited the most leading findings exemplified by impeding prominent inhibitory effects against pyocyanin, biofilm formation, twitching, and swarming motility by 75%, 91%, 76%, and 92%, respectively.

Even though our investigation presented promising findings, some limitations in our work should be well-framed. First, a lack of integrative study combining transcriptomics with metabolomics to fully unlock the core regulatory genes orchestrating the metabolic pathways, which in turn govern the chemical alterations during the different processing treatments. Secondly, blocking bacterial QS communication as an effective strategy to make bacteria more vulnerable to antibiotics and the immune system should be expounded on different pathogens, such as *Salmonella, Campylobacter, E. coli, and Listeria,* to strengthen the biological relevance. Thirdly, a lack of mechanistic studies beyond the QS inhibitory effect of seed extracts from a molecular aspect, dissecting the expression level of the core QS-related genes crucial for QS signalling and regulation in different pathogens. Fourthly, effective analytical platforms merging isolation, purification, functional modification, and mutual efficacy assessment for the biologically active compounds that might underlie the anti-QS potential from mustard sprouts are largely deficient. Undoubtedly, all these limitations should be considerably addressed in future research work to expand the impact of germination and roasting treatments on mustard seeds from chemical and functional standpoints, transforming the edible seeds into a valuable functional food source.

## Methods

### Chemicals and reagents

Chromatographic grade solvents, including methanol, acetonitrile and formic acid utilised in LC–MS analysis were acquired from Merck, Darmstadt, Germany. The reference standards: sinigrin, leucine, ferulic acid, kaempferol, linolenic acid and stigmasterol were from Sigma-Aldrich (St Louis, MO, USA).

### Seeds acquisition and preparation

In June 2023, brown mustard seeds were acquired from the Agricultural Research Centre at Cairo University in Egypt. A batch of uniformly sized seeds (150 g), were carefully cleaned and surface disinfected for 5 min using a 0.5% sodium hypochlorite solution and then thoroughly washed with distilled water to remove disinfectant residues. Next, the mustard seeds batch was equally divided into three portions, where the first portion was directly powdered with an electric blender, labelled with MS (C) (control mustard seeds) and stored at 4 °C prior to extraction. The second fraction was subjected to 10-day germination treatment following^4^ protocol with minor amendments. In summary, 50 g of seeds were placed on filter paper sheets in a biochemical container. The seeds were left in darkness at 25 ± 2 °C and 60% relative humidity to germinate. Using a mist generator, sterile water was sprayed twice a day to keep the seeds moist. After that, the sprouted seeds were lyophilised, powdered, sieved through a 0.5-mm mesh, labelled with MS (G) (germinated mustard seeds) and kept at 4 °C until further analysis.

The remaining set (50 g) was subjected to controlled roasting treatment following a previous protocol described by us with minor changes^16^. For roasting treatment, mustard seeds were spread out in uniform thin layers (2–3 mm thick) on 25 × 25 cm trays that had been preheated and placed in a preheated conventional oven (Nüve FNO55, Ankara, Turkey) at 180 °C for 10 min. Afterwards, the roasted seeds are allowed to cool in desiccators at room temperature for 1 h. The seeds were then powdered, sieved through a 0.5-mm mesh, labelled with MS (R) (roasted mustard seeds) and stored at 4 °C until further use.

### Extraction of differently treated mustard seeds

Next, the resulting portions of mustard seeds differently treated as illustrated above were submitted to the extraction process, aligning with our previously optimised methods^42,43^. Briefly, in an ultrasonic bath apparatus (3 L Alpha Plus, Japan), 50 grams of each resultant fraction were extracted twice separately in 200 mL of 70% ethanol at 40 °C for 60 min. The samples’ extracts were vacuum evaporated using a rotary evaporator (Buchi Rotavapor, Switzerland) until dryness. Five independent replicates of each treatment were used for further investigation.

### Phytochemical analysis of different mustard extracts using UPLC–QqQ–MS/MS

Chemical profiling of mustard seeds/sprouts extracts post exposure to different priming treatments was conducted using Shimadzu 8045 UPLC triple quadruple (QqQ) apparatus (Shimadzu corporation, Kyoto, Japan) integrated with an electrospray ionisation (ESI) source.

Under the guidance of ref. ^11^ protocol, a detailed description of chromatographic conditions, the operational parameters for ESI interface and mass data processing parameters was provided in the supplementary material.

Crucially, a detailed description of standard solutions preparation for UPLC–MS relative quantification and method validation has been assigned in the supplementary material, where the analytical parameters, including linearity, limit of detection (LOD), and limit of quantification (LOQ) were based on International Council for Harmonisation (ICH) criteria for analytical validation as complied in Table S3.

### Multivariate analysis of the UPLC–MS data and pathway enrichment analysis

Herein, the gathered raw UPLC–MS data were uploaded onto SIMCA-P software (version 14.1; Umetrics, Umea, Sweden) for chemometric analysis. Data overview tool, Principal Component Analysis (PCA), was preliminarily inserted for giving an overview mapping of the collinear dataset, revealing clusters, outliers and trends in the data. Complementarily, Orthogonal Projection to Latent Structures-Discriminant Analysis (OPLS-DA) was incorporated as a superior classification attempt to maximally illuminate confounding variations among the comparable mustard samples, steadily pinpointing metabolic signatures post germination and roasting treatments of mustard seeds. With a scope to catch differential metabolites post exposure to germination and roasting processes, OPLS-DA models and the respective volcano plots were conducted as a pairwise modelling approach between different treated seeds versus control (untreated) ones as follows; the pairwise comparisons were (MS (C) & MS (G)) and (MS (C) & MS (R)). The metabolites’ contributions to the change rates of *Y*-variables in OPLS-DA were characterised using variable importance of projection (VIP) values, where VIP > 1 suggested the notable dedication of these metabolites to group classifications. Furthermore, a one-way analysis of variance (ANOVA) followed by Tukey’s multiple range test was used to determine the statistical significance between concentration means at the different treatments, with a significance cutoff of *p* < 0.05 and fold-changes (FC) > 2.0 (or <0.5). Therefore, the compounds that satisfied the requirements of VIP > 1, FC > 2.0 (or <0.5), and *p*-values < 0.05 were ultimately selected as “differential metabolites.” Of note, the corresponding *R*^2^ and *Q*^2^ were used to evaluate the models’ prediction reliability.

MetaboAnalyst 6.0 (https://www.metaboanalyst.ca/) was exploited to analyse the different metabolites in each of the pairwise groups, where the heatmaps and respective volcano plots among the comparison samples were constructed. The Kyoto Encyclopaedia of Genes and Genomes (KEGG) database (https://www.metaboanalyst.ca/), plugged into MetaboAnalyst 6.0 software, was chosen for metabolic pathway enrichment analysis, where the metabolic pathway was considered to be significantly enriched if its impact value was greater than 0.1 and its *p*-value was less than 0.05.

### Evaluation of QS inhibitory activity of different mustard extracts

#### Preliminary antimicrobial screening

The different extracts of mustard seeds, control, germinated, and roasted, were initially screened for antimicrobial activity against *Pseudomonas aeruginosa* PAO1 using the well-diffusion method^44,45^. Ciprofloxacin served as the positive control. Each extract was dissolved in 20% (v/v) DMSO at a concentration of 5 mg/mL, and 50 μL was dispensed into 10 mm wells on Luria-Bertani (LB) agar plates. The diameter of the inhibition zones was measured to assess antibacterial activity.

### Minimum inhibitory concentration (MIC) and sub-MIC effects

The extracts exhibiting antimicrobial activity were further evaluated to determine their minimum inhibitory concentration (MIC) using the broth microdilution assay in a 96-well plate according to CLSI guidelines^46^. A volume of 100 μL of Mueller-Hinton broth was dispensed into each well, followed by the addition of 100 μL of each compound. Serial two-fold dilutions (ranging from 2500 to 5 μg/mL) were prepared. Each well was inoculated with *P. aeruginosa* PAO1 at ~5 × 10⁵ CFU/mL. Controls included untreated bacteria (positive) and media only (negative). Plates were incubated at 37 °C for 24 h, and the MIC was defined as the lowest concentration with no visible growth. Sub-inhibitory concentrations (sub-MICs) were also determined for subsequent analysis^47^.

### Growth curve analysis

The impact of sub-MICs of control and germinated seed extracts at ½ MIC on the growth of *P. aeruginosa* PAO1 was evaluated. Overnight cultures were inoculated into 100 mL of LB broth, treated with the test compounds, or left untreated, and incubated at 37 °C. Bacterial growth was monitored at 2-h intervals by measuring optical density at OD_600_^48^.

### Anti-virulence activity

The effect of the different extracts of mustard seeds, control, germinating, and roasted, was evaluated on virulence traits of *P. aeruginosa* PAO1, including biofilm formation, pyocyanin production, and motility. Control and germinating seed extracts were evaluated at ½ MIC. All assays were conducted in triplicate using culture supernatants^49^.

### Pyocyanin quantification

Pyocyanin production was measured after culturing *P. aeruginosa* PAO1 in King’s A broth (2% peptone, 1% K₂SO₄, 0.14% MgCl₂) at 150 rpm for 48 h. Pyocyanin was extracted as described by ref. ^50^, and concentrations (μg/mL) were determined by measuring absorbance at OD_520_ and applying the factor OD_520_ × 17.072.

### Motility assays

To assess twitching, swarming, and swimming motility, overnight cultures were diluted to an OD_600_ of 0.1–0.2. For twitching, 2 μL of the culture was stab-inoculated into 1% LB agar plates with or without compounds and incubated at 37 °C for 48 h. Zone diameters were recorded^51^. Swimming motility was assessed on agar plates containing 1% tryptone, 0.5% NaCl, and 0.5% agar. Treated and untreated plates were inoculated with 2 μL and incubated at 37 °C for 18 h^51,52^. Swarming motility was tested on agar plates containing 0.5% peptone, 0.2% yeast extract, 1.0% glucose, and 0.5% agar^53^. Plates were inoculated with 2 μL of the diluted PAO1 culture and incubated at 37 °C for 16 h^54^. All motility assays were conducted in triplicate using both treated and untreated samples.

### Biofilm inhibition assay

Biofilm formation was quantified using the crystal violet staining method^55^. Overnight cultures were diluted and incubated in 96-well microtiter plates containing LB with or without the test compounds for 24 h at 37 °C. After incubation, non-adherent cells were removed, and the wells were gently washed with 0.9% NaCl. The adherent biofilm was fixed with 99% methanol, stained with 0.5% (w/v) crystal violet, washed, and air-dried. The bound dye was solubilized in 33% (v/v) glacial acetic acid, and absorbance was measured at OD_492_. All tests were performed in triplicate^56^.

### Efficacy-directed discrimination of the different mustard extracts and bioactives discovery using OPLS analysis

Following that, the orthogonal projection to latent structures (OPLS) model was incorporated as a biologically relevant classification model to glimpse the principal biologically active compounds that might underlie the observable QS inhibitory activity of the different investigated mustard extracts. Notably, *R*^2^ cum and *Q*^2^ cum were used to evaluate the quality of the established models, where excellent models had values near 1.

### Statistical analysis

Data were analysed using Excel’s Data Analysis Toolpak. Means and standard deviations were calculated, and statistical significance was evaluated using Welch’s t-test with thresholds set at *p ≤ 0.05 and **p ≤ 0.01. Each value represents the mean of three independent replicates.

## Supplementary information

Supplementary Information

## Data Availability

Data will be made available on request.
