# Supplementary material for: Comparison of metabolic profiles, regulatory pathways, and quorum sensing inhibition activity of brown mustard seeds (Brassica juncea L.) post germination and roasting treatments
Source: NPJ Sci Food. 2025 Nov 11;9:221. doi: 10.1038/s41538-025-00606-5 (PMC12606101; doi:10.1038/s41538-025-00606-5)
Supplement: Supplementary file 1 — Supplementary Information [file 41538_2025_606_MOESM1_ESM.pdf]

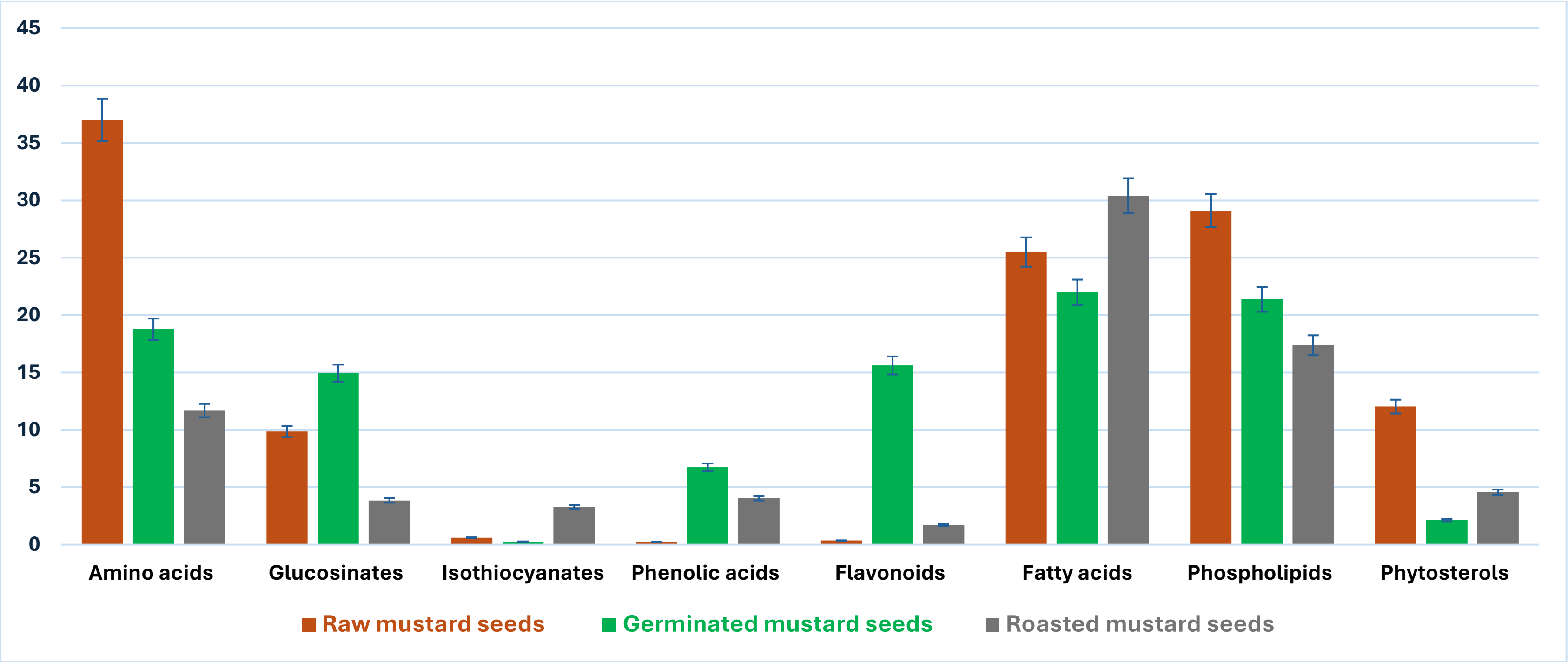

Figure S1: **The relative average content of the main chemical classes in the mustard samples (data are expressed as mg standard equivalent per gram of dry extract).**

Table S1: List of differential metabolites between raw and germinated mustard seeds

Compound name	VIP	FC Germinated /raw (control)	P value	Trend MS (G) / MS (C)	Related metabolic pathway
Linolenic acid	1.24153	106.75	1.00E-06	Up	alpha-Linolenic acid metabolism
Valyl-Phenylalanine	1.14	0.003377	2.00E-03	Down	Phenylalanine, tyrosine and tryptophan biosynthesis
Isorhamnetin	1.08153	90.539	1.00E-05	Up	Flavonoid biosynthesis
Hydroxy arachidonic acid	1.07153	80.611	3.00E-04	Up	Arachidonic acid metabolism
Stearidonoyl-Glycerol	1.06153	70.436	3.00E-04	Up	Glycerophospholipid metabolism
Gluconasturtiin	1.05153	0.015445	3.00E-03	Down	Sulphur metabolism
LysoPC (16:0)	1.041	0.026034	2.00E-02	Down	Glycerophospholipid metabolism
Ricinoleic acid	1.04	22.827	4.00E-04	Up	Biosynthesis of unsaturated fatty acids
Myristic acid	1.04	0.06225	4.00E-02	Down	Fatty acid biosynthesis
Eicosenoic acid	1.03153	0.091083	1.00E-02	Down	Fatty acid biosynthesis
Leucine	1.04153	10.964	5.00E-02	Up	Glycine, serine and threonine metabolism
Nonanedioic acid (Azelaic acid)	1.04153	0.099638	1.00E-03	Down	Fatty acid biosynthesis
Butyl glucosinolate	1.04153	0.12346	4.00E-02	Down	Sulphur metabolism
Carnosic acid	1.04153	0.13695	4.00E-02	Down	Steroid biosynthesis

Syringic acid	1.04153	7.1314	8.00E-04	Up	Phenylpropanoid biosynthesis
Sinapine	1.042	6	2.00E-03	Up	Phenylpropanoid biosynthesis
Indole-3-carbinol	1.04	6	3.00E-02	Up	Phenylpropanoid biosynthesis
Coumaric acid	1.03	6	3.00E-02	Up	Phenylpropanoid biosynthesis
Hydroxyquinoline	1.05	6	3.00E-03	Up	Phenylpropanoid biosynthesis
4-Hydroxybenzoic acid	1.04	6	1.00E-04	Up	Phenylpropanoid biosynthesis
Dihydrosinapicacid	1.03	6	2.00E-02	Up	Phenylpropanoid biosynthesis
Salicylic acid-O-hexoside	1.0432	6	4.00E-03	Up	Phenylpropanoid biosynthesis
vanillic acid	1.04108	6	4.00E-02	Up	Phenylpropanoid biosynthesis
Sinapic acid	1.04109	6	1.00E-02	Up	Phenylpropanoid biosynthesis
MethylCoumarin	1.04096	6	5.00E-02	Up	Phenylpropanoid biosynthesis
Ellagic acid	1.04084	6	1.00E-03	Up	Phenylpropanoid biosynthesis
Chlorogenic acid (3-O-Caffeoylquinic acid)	1.04083	6	4.00E-02	Up	Phenylpropanoid biosynthesis
Epicatechin	1.04047	6	4.00E-02	Up	Flavonoid biosynthesis
Astringin	1.04047	6	4.00E-03	Up	Stilbenoid, diarylheptanoid and gingerol biosynthesis
luteolin-4'-O-glucoside	1.04047	6	4.00E-02	Up	Flavonoid biosynthesis
Syringetin-3-O-glucoside	1.03978	6	5.00E-02	Up	Flavonoid biosynthesis

Coumaroyltartaric acid	1.03962	6	1.00E-03	Up	Phenylpropanoid biosynthesis
Sinalbin A	1.03929	6	1.00E-05	Up	Phenylpropanoid biosynthesis
Quercetin	1.03929	6	2.00E-03	Up	Flavonoid biosynthesis
Daidzein	1.03871	6	1.00E-04	Up	Flavonoid biosynthesis
Hydroxy palmitic acid	1.03854	6	3.00E-02	Up	Fatty acid biosynthesis
Hydroxy linolenic acid	1.03783	6	3.00E-02	Up	Biosynthesis of unsaturated fatty acids
Linolenoyl-glycerol	1.0361	6	1.00E-04	Up	Glycerophospholipid metabolism
Linoleoyl glycerol	1.0361	6	2.00E-02	Up	Glycerophospholipid metabolism
Phenylalanine	1.03578	5.9365	4.00E-04	Up	Phenylalanine metabolism
GABA	1.03532	0.172	4.00E-02	Down	Alanine, aspartate and glutamate metabolism
gamma-glutamyl leucine	1.03445	0.172	1.00E-02	Down	Alanine, aspartate and glutamate metabolism
Acetyl-L-tyrosine	1.0343	0.172	5.00E-02	Down	Phenylalanine, tyrosine and tryptophan biosynthesis
Tryptamine	1.03373	0.172	1.00E-03	Down	Phenylalanine, tyrosine and tryptophan biosynthesis
Dodecanoic acid (Lauric acid)	1.03153	0.172	4.00E-02	Down	Fatty acid biosynthesis
PE (12:0/16:0)	1.03139	0.172	4.00E-02	Down	Glycerophospholipid metabolism

Stearidionic acid	1.03118	0.172	8.00E-04	Down	Biosynthesis of unsaturated fatty acids
Feruloyltryptamine	1.03097	0.17511	1.00E-05	Down	Indole alkaloid biosynthesis
Glycylvaline	1.03059	5.3548	2.00E-03	Up	Glycine, serine and threonine metabolism
PG (14:0/14:0)	1.02989	0.19223	1.00E-04	Down	Glycerophospholipid metabolism
Peonidin-O-glucoside	1.02928	4.8024	3.00E-02	Up	Flavonoid biosynthesis
glucobrassicin	1.01952	4.297	3.00E-02	Up	Glucosinolate biosynthesis
Serine	1.04153	0.30057	3.00E-03	Down	Glycine, serine and threonine metabolism
kaempferol	1.04153	3.1813	1.00E-04	Up	Flavonoid biosynthesis
Progoitrin	1.03984	3.1	1.00E-02	Up	Glucosinolate biosynthesis
Sinigrin	1.04153	2.9432	2.00E-02	Up	Glucosinolate biosynthesis
Erucic acid	1.04153	2.4965	4.00E-03	Up	Fatty acid biosynthesis
Glucocheirolin	1.04153	2.3	1.00E-03	Up	Glucosinolate biosynthesis
Gluconapin	1.041	2.3	1.00E-03	Up	Glucosinolate biosynthesis

Table S2: List of differential metabolites between raw and roasted mustard seeds

Compound name	VIP	FC Roasted /raw (control)	P value	Trend MS (R) / MS (C)	Related metabolic pathway
Erucic acid	1.14153	184.76	1.00E-05	Up	Fatty acid biosynthesis
L-Phenylalanine	1.14	0.006454	2.00E-03	Down	Phenylalanine metabolism
Isorhamnetin	1.07153	35.171	1.00E-04	Up	Flavonoid biosynthesis
GABA	1.06153	0.02756	3.00E-02	Down	Alanine, aspartate and glutamate metabolism
Linolenic acid	1.05159	24.983	3.00E-02	Up	Biosynthesis of unsaturated fatty acids
1-palmitoyl-sulfoquinovopyranosylglycerol SQMG(16:0)	1.05153	0.044738	3.00E-03	Down	Sulphur metabolism
L-Valyl-L-Phenylalanine	1.04153	0.044978	1.00E-04	Down	Phenylalanine metabolism
Glucobrassicin	1.041	0.159	2.00E-02	Down	Sulphur metabolism
gamma-glutamylleucine	1.04	0.048619	4.00E-04	Down	Alanine, aspartate and glutamate metabolism
Gluconasturtiin	1.04	0.050426	4.00E-02	Down	Sulphur metabolism

Beta-tocopherol	1.03153	0.061015	1.00E-02	Down	Ubiquinone and other terpenoid-quinone biosynthesis
Hydroxy lauric acid	1.04153	0.10164	5.00E-02	Down	Fatty acid biosynthesis
Hydroxy arachidonic acid	1.04153	9.7676	1.00E-03	Up	Biosynthesis of unsaturated fatty acids
Tryptamine	1.04153	0.10688	4.00E-02	Down	Indole alkaloid biosynthesis
Allyl isothiocyanate	1.04153	9.2636	4.00E-02	Up	Sulphur metabolism
Desmosterol	1.04153	0.10879	8.00E-04	Down	Steroid biosynthesis
Myristic acid	1.04153	0.11711	1.00E-05	Down	Fatty acid biosynthesis
Carnosic acid	1.042	0.12035	2.00E-03	Down	Ubiquinone and other terpenoid-quinone biosynthesis
N-Feruloyltryptamine	1.041	0.15785	1.00E-04	Down	Indole alkaloid biosynthesis
N-Acetyl-L-tyrosine	1.04	0.172	3.00E-02	Down	Phenylalanine metabolism
Hydroxyglucobrassicin	1.03	0.172	3.00E-02	Down	Sulphur metabolism
Stigmasterol	1.05	0.172	3.00E-03	Down	Steroid biosynthesis

PE (12:0/16:0)	1.04	0.172	1.00E-04	Down	Glycerophospholipid metabolism
Stearidionic acid	1.03	0.172	2.00E-02	Down	Biosynthesis of unsaturated fatty acids
1-Stearidonoyl-Glycerol	1.0432	0.172	4.00E-03	Down	Glycerophospholipid metabolism
Glucocheirolin	1.04108	0.6235	4.00E-02	Down	Sulphur metabolism
Sinapine	1.04109	5.6235	1.00E-02	Up	Phenylpropanoid biosynthesis
Gluconapin	1.04096	0.235	5.00E-02	Down	Sulphur metabolism
Indole-3-carbinol	1.04084	5.6235	1.00E-03	Up	Phenylpropanoid biosynthesis
Phenylethyl isothiocyanate	1.04083	5.6235	4.00E-02	Up	Sulphur metabolism
4-Hydroxybenzoic acid	1.04047	5.6235	4.00E-02	Up	Phenylpropanoid biosynthesis
Dihydrosinapic acid	1.04108	5.6235	4.00E-03	Up	Phenylpropanoid biosynthesis
Vanillic acid	1.04109	5.6235	4.00E-02	Up	Phenylpropanoid biosynthesis
Sinapic acid	1.04096	5.6235	1.00E-02	Up	Phenylpropanoid biosynthesis

benzyl isothiocyanate	1.04084	5.6235	5.00E-02	Up	Sulphur metabolism
Canolol	1.04083	5.6235	1.00E-03	Up	Sulphur metabolism
Chlorogenic acid (3-O-Caffeoylquinic acid)	1.04047	5.6235	1.00E-05	Up	Phenylpropanoid biosynthesis
Luteolin-4'-O-glucoside	1.03047	5.6235	2.00E-03	Up	Flavonoid biosynthesis
Progoitrin	1.03047	0.3235	1.00E-04	Down	Sulphur metabolism
Syringetin-3-O-glucoside	1.02047	5.6235	3.00E-02	Up	Flavonoid biosynthesis
Glucotropaeolin	1.0204	0.6235	3.00E-02	Down	Sulphur metabolism
Hydroxy palmitic acid	1.02	5.6235	1.00E-04	Up	Fatty acid biosynthesis
Hydroxy linolenic acid	1.02	5.6235	2.00E-02	Up	Biosynthesis of unsaturated fatty acids
Lyso PC 18:3	1.02	5.6235	4.00E-04	Up	Glycerophospholipid metabolism
Linolenoyl-glycerol	1.02	3.6235	4.00E-02	Up	Glycerophospholipid metabolism
Linoleoyl glycerol	1.02	3.6235	1.00E-02	Up	Glycerophospholipid metabolism

Leucine	1.02	0.20579	5.00E-02	Down	Glycine, serine and threonine metabolism
Sinigrin	1.02	4.063	1.00E-03	Up	Glucosinolate biosynthesis
PG (14:0/14:0)	1.02	0.25769	4.00E-02	Down	Glycerophospholipid metabolism
Nonanedioic acid (Azelaic acid)	1.02	0.2884	4.00E-02	Down	Fatty acid biosynthesis
Stearic acid	1.017	3.3972	8.00E-04	Up	Fatty acid biosynthesis
Lyso PC (16:0)	1.017	0.32632	1.00E-05	Down	Glycerophospholipid metabolism
Ferulic acid	1.017	2.6797	2.00E-03	Up	Phenylpropanoid biosynthesis
Ricinoleic acid	1.017	0.37745	1.00E-04	Down	Biosynthesis of unsaturated fatty acids
Glycylvaline	1.016	0.43395	3.00E-02	Down	Glycine, serine and threonine metabolism
Eicosapentanoic acid	1.013	0.4652	3.00E-02	Down	Biosynthesis of unsaturated fatty acids

Standard solutions preparation for UPLC-MS relative quantification

All characterized components in differently processed mustard extracts were relatively quantified based on mean peak area computation using the calibration curves of respective standards and the gained results were readily expressed as (mg standard equivalents /g dry extract). Sinigrin, leucine, ferulic acid, kaempferol, linolenic acid, and stigmasterol were prepared as stock external standard solutions and the result were readily presented as (mg standard equivalents /g dry extract). The stock external standard solution of the reference standards was prepared as follows: an accurate weight of each standard (10 mg) was separately placed in 10-mL volumetric flask. HPLC-grade methanol was added, and the solution was serially diluted to the working concentrations over the reliable range 0.0125 – 0.75 mg/ ml using the same solvent. Five µL aliquots of each standard compound were applied onto the chromatographic column and the injections were performed in duplicates for different concentration levels. The standard calibration curves were established by plotting peak areas of the standards as the analytical responses against their known concentration concentrations. Parameters like linearity, limit of detection (LOD) and limit of quantification (LOQ) were assessed based on ICH guideline on bioanalytical method validation [1].

Phytochemical analysis of different mustard extracts using UPLC-MS/MS

Metabolic profiling of different mustard extracts was performed on a Shimadzu 8045 UPLC triple quadruple (QqQ) apparatus (Shimadzu corporation, Kyoto, Japan) coupled with an electrospray ionization (ESI) source. The apparatus is equipped with a pump (LC 2040), an autosampler (SIL-30AC), a detector (LC-2030/2040 PDA detector), and an on-line degasser. Chromatographic separation was conducted using a Shimpack GISS UPLC C18 column (50 mm × 2.1 mm ID × 1.7 µm particle size) (Shimadzu corporation, Kyoto, Japan) maintained at 30°C. The biphasic mobile phase consisted of acidified ultrapure water (0.1% formic acid) (Phase A) and acetonitrile (Phase B) was gradient eluted at a flow rate of 0.2 mL /min and programmed as follows: 5% B at retention time of 0.0–10.0 min, 40% B at 10.0–14.0 min, 50% B at 14.0–18.0 min, 70% B at 18.0–22.0 min, 90% B at 22.0–26.0 min, 95% at 26.0–30.0 min, 100% B at 30.0–35.0 min and finally returning to the initial condition at 35 min. The suitably chosen set of operational parameters for ESI interface operated in both positive and negative ion modes with full scan function covering the mass range of 50–1200 Da were set according to our previous publication [2] as follows: capillary voltage of 3 kV, cone voltage; 35 V, the ion source temperature was 150◦C, the nebulizer (nitrogen gas) pressure was 35 psi, drying and sheath gas (N_2) temperature was 440 ˚C and 350 ˚C, respectively. The drying and sheath gas

flows were applied at 900 L/h and 50 L/h, respectively. The scan time and interscan delay were set to 0.4 s and 0.1 s, respectively and the analytical run time was extended to 35 min.

The data processing was conducted using MZmine 2.0 analysis software. The processing parameters were set as follows: mass range 100–1000 Da, mass tolerance 0.15 Da, RT tolerance (min) 0.2, signal-to-noise threshold 1.5. Afterwards, a data matrix compressing the retention time, mass-to-charge ratio (m/z) and normalized peak area was generated. Of note, metabolite annotations were established according to retention times relative to external standards. As well, our in-house database, tandem mass spectra (quasi-molecular ions coupled with distinctive MS/MS fragmentation patterns), relevant reference literature data and dictionary of natural products database (DNP) were all utilized to present high confidence level of annotation according to Metabolomics Standards Initiative (MSI) criteria. Of note, 5 µl from each sample were pooled to create quality control (QC) samples for assessing the reproducibility and robustness of the analytical platform.

Table S3: Linearity and sensitivity parameters for standard compounds

Compound	Linearity range (mg /ml)	Slope (a)	Intercept (b)	Correlation coefficient (r)	LOD (mg /ml)	LOQ (mg /ml)
Sinigrin *	0.013-0.36	$3.34*10^7$	$-1.05*10^4$	0.98	0.004	0.03
Leucine **	0.01-0.4	$6.27*10^7$	$-4.12*10^3$	0.99	0.006	0.02
Ferulic acid***	0.02-0.6	$7.5*10^7$	$-2.15*10^3$	0.992	0.008	0.03
kaempferol ****	0.021-0.64	$6.45*10^7$	$-1.65*10^4$	0.994	0.009	0.04
linolenic acid *****	0.022-0.8	$2.25*10^7$	$-7.41*10^4$	0.996	0.014	0.07
Stigmasterol *******	0.025-0.5	$1.25*10^7$	$-3.81*10^3$	0.98	0.01	0.06

For each calibration curve the equation is y= ax + b, where y is the peak area, x is the concentration of the standard (mg/ml), a is the slope, b is the intercept, r the correlation coefficient, LOD is the limit of detection and LOQ is the limit of quantitation.

*** Used for semi-quantitative analysis of glucosinolates.**

**** Used for semi-quantitative analysis of f amino acids.**

***** Used for semi-quantitative analysis of phenolic acids**

****** Used for semi-quantitative analysis of flavonoids.**

******* Used for semi-quantitative analysis of fatty acids and their derivatives.**

********* Used for semi-quantitative analysis of terpenoids and phytosterols.**

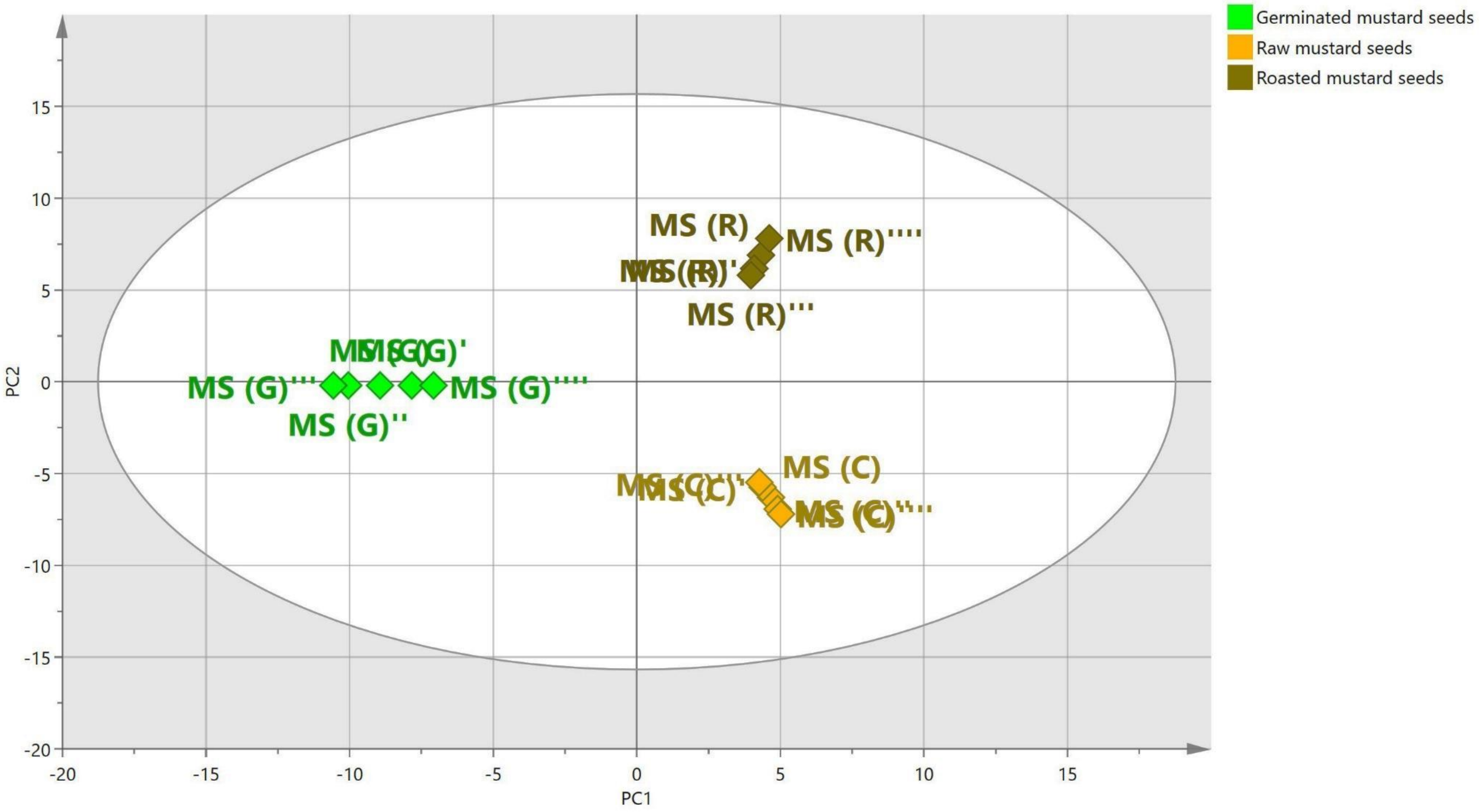

Figure S2: PCA score plot of different mustard samples

References

1. Kadian, N. *et al.* Comparative assessment of bioanalytical method validation guidelines for pharmaceutical industry. *J. Pharm. Biomed. Anal.* **126**, 83–97 (2016).

2. Ghallab, D. S., Mohyeldin, M. M., Shawky, E., Metwally, A. M. & Ibrahim, R. S. Chemical profiling of Egyptian propolis and determination of its xanthine oxidase inhibitory properties using UPLC–MS/MS and chemometrics. *Lwt* **136**, 110298 (2021).
